# Adaptive fault-tolerant control of mixed-order vehicles platoon with actuator saturation and disturbances

**DOI:** 10.1038/s41598-026-49842-z

**Published:** 2026-05-12

**Authors:** Minghao Yang, Yiguang Wang, Xiaojie Li

**Affiliations:** 1 Key Laboratory of Advanced Manufacturing and Automation Technology, Guilin University of Technology, Guilin, 541006 China; 2 Guangxi Engineering Research Center of Intelligent Rubber Equipment, Guilin University of Technology, Guilin, 541006 China; 3 College of Mechanical and Control Engineering, Guilin University of Technology, Guilin, 541006 China

**Keywords:** Connected and autonomous vehicles (CAVs) platoon, Mixed-order dynamics, Fault-tolerant control, Neural networks (NNs), Disturbance observer, Actuator saturation, Engineering, Mathematics and computing, Physics

## Abstract

This paper investigates the distributed adaptive fault-tolerant control problem for mixed-order connected and autonomous vehicles (CAVs) platoon with actuator faults, saturation, external disturbances, and uncertainties. The studied mixed-order CAVs platoon consists of vehicles with mixed second- and third-order dynamics and all vehicles can have the different numbers and types of states. As the actuator’s efficiency may dynamically fluctuate due to the degrees of wear-outs, aging, and overheating of the actuator during the entire operational process, it is more practical to consider the actuator faults to be nonlinear and time-varying rather than constant. A new adaptive disturbance observer (ADOB) is designed to approximate external disturbances as well as bias faults and improve the control performance of mixed-order CAVs platoon. The Neural networks (NNs)-based adaptive mechanism is designed to approximate uncertainties and actuator efficiency factor, which can mitigate the adverse effects of uncertainties as well as actuator faults and enhance the system’s robustness. For the mixed-order CAVs platoon with actuator faults, actuator saturation, external disturbances, and uncertainties, a novel adaptive fault-tolerant control method based on the developed ADOB and NNs adaptive mechanism is proposed to ensure stability and achieve the control goals of the mixed-order CAVs platoon. The numerical example is provided to demonstrate the effectiveness and advantage of the developed control method.

## Introduction

Recently, with the dramatic increase in the number of automobiles, the problems such as traffic congestion, environmental pollution, and driving safety have become the primary traffic challenges^1–5^. Vehicle platoon control technology for connected and autonomous vehicles (CAVs) offers a promising solution for alleviating traffic congestion, reducing fuel consumption, enhancing driving safety, and improving traffic efficiency^6^. Many issues in platoon control have been studied, such as communication topology^7^, actuator saturation^8^, and actuator faults^9^, etc.

The main objective of CAVs platoon control is to ensure that all following vehicles can track the leader’s velocity, while maintaining the desired inter-vehicle spacing. Based on the analysis of existing literature, there are two classes of vehicle models widely used in the field of CAVs platoon: one is the second-order vehicle dynamics of Position-Velocity, and the other is the third-order vehicle dynamics of Position-Velocity-Acceleration^10–12^. In^10^, for the third-order vehicle dynamics, a robust control method based on the Kalman filter is developed to compensate for the adverse impact of external disturbances and achieve the control goals of CAVs platoon. In^11^, a distributed model predictive control approach for CAVs platoon with second-order vehicle dynamics under abnormal communication conditions is designed to maintain the desired inter-vehicle spacing and ensure consistent velocity and acceleration. In^12^, Based on the third-order vehicle dynamics, a disturbance observer (DOB)-based finite-time sliding mode control (SMC) is proposed to achieve the control goals of CAVs platoon. In practical applications, due to the fact that diverse tasks may require to be accomplished by various kinds of vehicles, the CAVs platoon may simultaneously contain vehicles with second- and third-order dynamics^13^, which is called the mixed-order CAVs platoon^14^. However, the existing studies have primarily focused on single-type vehicle models (second- or third-order CAVs platoon), which limits the applicability of CAVs platoon in more practical control scenarios. Therefore, in order to expand the application scope of the CAVs platoon, it is of great significance and more challenging to further research control issues of the mixed-order CAVs platoon. This is because mixed-order CAVs platoon better reflect the real-world traffic, where diverse vehicle types coexist in complex driving environments.

In CAVs platoon, the vehicles are inevitably affected by external disturbances, which may lead to a decline of control performance or even instability for the CAVs^15^. Numerous methods have been proposed to address external disturbances and uncertainties of CAVs^15–17^. In^15^, an adaptive integral SMC approach based on nonlinear DOB is proposed to handle external disturbances and ensure that the tracking error of CAVs is finite-time bounded. In^16^, the external disturbances are estimated by using the fixed-time DOB to mitigate the adverse effects of external disturbances, and a terminal SMC is proposed for CAVs platoon to ensure that following vehicles can track the leader. In^17^, a novel DOB is designed to estimate and compensate for external disturbances within a prescribed time, and a prescribed-time SMC is proposed to achieve the control goals for CAVs platoon. However, it is worth noting that the prior knowledge of the upper bounds about the external disturbances or their first derivatives is required in above-mentioned DOB-based control methods. In practice, due to wind resistance, complex road conditions, and parameter uncertainties, it is often difficult or impossible to obtain these precise upper bounds for CAVs platoon, which greatly limits the practical application range of these control methods.

In CAVs platoon, actuators faults usually occur in practice due to wear-outs, aging, and overheating, which may lead to a partial loss of effectiveness (indicated by actuator efficiency factor) and bias faults^18–25^. In order to address the problem of actuator faults, some methods have been proposed to reduce the adverse effects of actuator faults and improve the system’s performance^26–28^. In^26^, a distributed fault-tolerant control approach based on nonlinear DOB is proposed for the CAVs platoon to estimate and compensate for actuator faults. In^27^, a robust adaptive sliding mode resilient control method is designed to ensure that the CAVs platoon recovers to stable states in finite time even if actuator faults occur. It is worth noting that the aforementioned fault-tolerant control strategies assume the actuator faults to be constant, whereas in practice, as the degrees of wear-outs, aging, and overheating of actuators may be changing throughout the entire operational process of the CAVs platoon, the actuator efficiency factor is usually nonlinear, time-varying and unknown. Therefore, it is necessary to design a new control method to address nonlinear, time-varying and unknown actuator faults for mixed-order CAVs platoon and improve the control performance. Addressing this problem is critical for enhancing the practical applicability and theoretical robustness of CAVs platoon control in complex traffic environments.

In light of the above analysis, this paper investigates the distributed adaptive fault-tolerant control problem for mixed-order CAVs platoon with actuator faults and saturation, external disturbances, and uncertainties. The studied mixed-order CAVs platoon consists of vehicles with mixed second- and third-order dynamics and all vehicles can have the different numbers and types of states. A novel adaptive disturbance observer (ADOB) is designed to approximate external disturbances as well as bias faults and improve the control performance of mixed-order CAVs platoon. The Neural networks (NNs)-based adaptive mechanism is designed to approximate uncertainties and actuator efficiency factor, which can mitigate the adverse effects of uncertainties as well as actuator faults and enhance the system’s robustness. Based on the developed ADOB and NNs adaptive mechanism, a novel adaptive fault-tolerant control method for mixed-order (second- and third-order) CAVs platoon is proposed to ensure stability and achieve the control goals. The main contributions of this paper are summarized as follows.

1. Unlike the existing studies of the CAVs platoon that predominantly adopt single-type vehicle models (second- or third-order CAVs platoon) with the same number and type of states^10–12^, this paper investigates the platoon control problem of mixed-order CAVs, where all vehicles can have different numbers and types of states, which renders the proposed method more general and broadens the application scope of CAVs platoon.
2. Compared with the DOBs^15–17^ that require the prior bound knowledge of external disturbances or their first derivatives, this article proposes a novel ADOB capable of adaptively estimating the upper bounds of external disturbances without requiring its prior bound knowledge, which can improve the control performance of mixed-order CAVs platoon.
3. A NNs-based adaptive update mechanism for mixed-order CAVs platoon is proposed to approximate the nonlinear, time-varying, and unknown actuator faults, which can effectively mitigate the adverse impacts of actuator faults and enhance the system’s robustness.
4. A novel adaptive fault-tolerant control method based on the developed ADOB and NNs is proposed for mixed-order CAVs platoon with actuator faults, saturation, external disturbances, and uncertainties to ensure stability and achieve the control goals of the mixed-order CAVs platoon.

The article’s structure is outlined as follows. The literature review is presented in Sect. Literature review. A brief introduction to graph theory and notations are presented in Sect. Graph theory. Mixed-order CAVs modeling and problem formulation are given in Sect. Mixed-order CAVs platoon modeling and problem formulation. The distributed adaptive fault-tolerant design and stability analysis are given in Sect. Distributed adaptive fault-tolerant control design and stability analysis. The numerical example is presented in Sect. Numerical example. The conclusion is drawn in Sect. Conclusion.

## Literature review

Beyond the aforementioned perspectives, numerous studies have examined various other aspects and techniques for CAVs platoon, and Table 1 summarizes the main drawbacks of these approaches.

In^28^, a distributed adaptive finite-time fault-tolerant approach is proposed for CAVs platoon to maintain consistent speed and desired spacing. The actuator fault is estimated by employing a distributed finite-time observer and adaptive fault parameter estimation law, which can mitigate the adverse effects of actuator faults.

In^29^, a fault-tolerant control framework is presented for second-order heterogeneous vehicle platoon that ensures robustness against measurement noise. The framework integrates fault estimation with robust control to mitigate the impact of faults and disturbances, which enhances the reliability and safety of vehicle platoon operations.

In^30^, a hierarchical prescribed performance control method is presented for CAVs platoon with actuator saturation to ensure that vehicles can track the obtained reference trajectory while avoiding inter- and intra-platoon collisions. The simulation results show that the safe lane-changing maneuver is achieved with the preset tracking accuracy.

In^31^, an adaptive integral sliding mode control approach is developed for CAVs platoon to accomplish the control objectives. By designing a tunnel prescribed performance control method, the tracking error converges within a prescribed region while enhancing system performance.

In^32^, the optimal integral sliding mode control problem is investigated for CAVs platoon with directed communication topologies and external disturbances. By applying finite time control theory, an integral sliding mode control approach is developed to guarantee that error converges within finite time.

In^33^, a distributed fault-tolerant consensus control protocol is proposed for third-order homogeneous vehicle platoon with cyber-physical threats and actuator faults. The co-designed framework incorporates decentralized fault-estimation unknown input observers and event-triggered distributed fault-tolerant consensus controllers, which can maintain the tolerance of consensus errors and ensure safe spacing.

In^34^, the sliding mode control control issue is studied for vehicle platoon with uncertainty and external disturbances. A multi power reaching law is introduced to alleviate the chattering problem and increase convergence velocity, and a DOB is employed to estimate external disturbances.

Based on the review of existing approaches summarized in Table 1, several limitations remain unaddressed. Many of the discussed control strategies rely on idealized assumptions, such as constant actuator efficiency factors, the absence of actuator saturation, single-type vehicle model, and prior bound knowledge, which restricts their applicability under more realistic operating conditions. While some methods address specific scenarios such as fault and external disturbance estimation, they often lack a unified framework that simultaneously handles multiple practical issues, including actuator faults, saturation, external disturbances, and uncertainties. To address these research gaps, this paper proposes a novel adaptive fault-tolerant control method based on the developed ADOB and NNs for mixed-order CAVs platoon with actuator faults, saturation, external disturbances, and uncertainties in order to ensure stability and achieve the control goals of the mixed-order CAVs platoon.

**Table 1:** Limitations of existing approaches.

References	Control Strategy	Model	Focus	Limitations
^28^	Finite-time fault-tolerant control	Second-order vehicle model	Fault detection and finite-time control	Limited to actuator efficiency factor being constant
^29^	Fault-tolerant control	Second-order vehicle model	Robustness and compensation of faults and disturbances	Limited to bias fault, actuator efficiency factor not addressed
^30^	Prescribed performance control	Third-order vehicle model	Prescribed performance and collision avoidance	No actuator failure analysis, scalability limitations
^31^	Sliding mode control	Second-order vehicle model	Tracking error and prescribed performance	No actuator failure and saturation analysis, applicability limitations
^32^	Optimal integral sliding mode control	Second-order vehicle model	Communication topology and control optimization	Only applicable to second-order vehicle platoon scenarios
^33^	Event-triggered distributed fault-tolerant control	Third-order vehicle model	Tolerance of actuator faults and cyber-physical threats	Only applicable to third-order vehicle platoon scenarios
^34^	Coupled sliding mode control	Second-order vehicle model	Estimation of uncertainty and external disturbances	Requires the prior bound knowledge of external perturbations

## Graph theory

Graph theory is introduced to define information communication between vehicles. For CAVs, each vehicle is defined as node $i\in \{1,2,\ldots ,m\}$. A communication topology of CAVs platoon having *m* nodes is denoted by graph ${\mathcal {G}}=({\mathcal {V}},{\mathcal {E}})$ where ${\mathcal {V}}=\{\mathcal {V}_{1}, \mathcal {V}_{2}, \ldots ,\mathcal {V}_{m}\}$ denotes the node set and ${\mathcal {E}}\subseteq {\mathcal {V}}\times {\mathcal {V}}$ represents the edge set. An edge from $\mathcal {V}_{i}$ to $\mathcal {V}_{j}$ is defined as $(\mathcal {V}_{i}, \mathcal {V}_{j}) \in {\mathcal {E}}$. The adjacency matrix $\boldsymbol{A} = \left[ a_{ij}\right]$ is used to represent the weighted directed digraph ${\mathcal {G}}$, where $a_{ij}>0$ if $(\mathcal {V}_{i}, \mathcal {V}_{j}) \in {\mathcal {E}}$ and otherwise $a_{ij}=0$. Define $d_i = \sum _{j=1}^{m} a_{ij}$ as the in-degree for node *i* and define $\boldsymbol{D}={diag}\left\{ d_1, d_2, \ldots , d_m\right\}$ as the in-degree matrix. The Laplacian matrix is defined as $\boldsymbol{L}=\boldsymbol{D}-\boldsymbol{A}$. We assume that the topology of digraph ${\mathcal {G}}$ is fixed in this study. There exists a state-based spanning tree if the graph contains an information path of specific state from the leader to the followers^1^. The directional links between the leader and followers are often described by the pinning matrix $\boldsymbol{B}={diag}\left\{ b_1, b_2, \ldots , b_m\right\}$, where $b_i > 0$ for $\forall i\in M=\{1,2,\ldots ,m\}$ if the edge from the leader to node *i* exists, and $b_i = 0$ otherwise.

*Notations:* $\mathbb {R}^n$ denotes the real *n* vector, $\mathbb {R}^{m\times n}$ denotes the real $m\times n$ matrix, ${\textbf {0}}_{m\times n}$ denotes the zero matrix in $\mathbb {R}^{m\times n}$, ${\textbf {0}}_{m}$ denotes the zero matrix in $\mathbb {R}^{m\times m}$, $\boldsymbol{I}_n$ denotes the identity matrix in $\mathbb {R}^{n\times n}$, ${\textbf {1}}$ denotes the unity vector $[1,1,\ldots ,1]^T$, ${\textbf {1}}_n$ denotes *n*-dimensional unit column vector, $\sigma (\bullet )$ denotes a matrix’s set of singular values, ${\overline{\sigma }}(\bullet )$ and ${\underline{\sigma }}(\bullet )$ are the maximum and minimum singular values, $tr\{\bullet \}$ denotes a matrix’s trace, $diag\{\bullet \}$ denotes the diagonal matrix, $|\bullet |$ represents the absolute value, $\Vert \bullet \Vert$ represents the Euclidean norm, $\Vert \bullet \Vert _F$ is the Frobenius norm; For any matrix $\boldsymbol{A}$, $\boldsymbol{A}>0$ denotes $\boldsymbol{A}$ is a positive definite matrix.

## Mixed-order CAVs platoon modeling and problem formulation

### Mixed-order CAVs platoon model

**Fig. 1:**
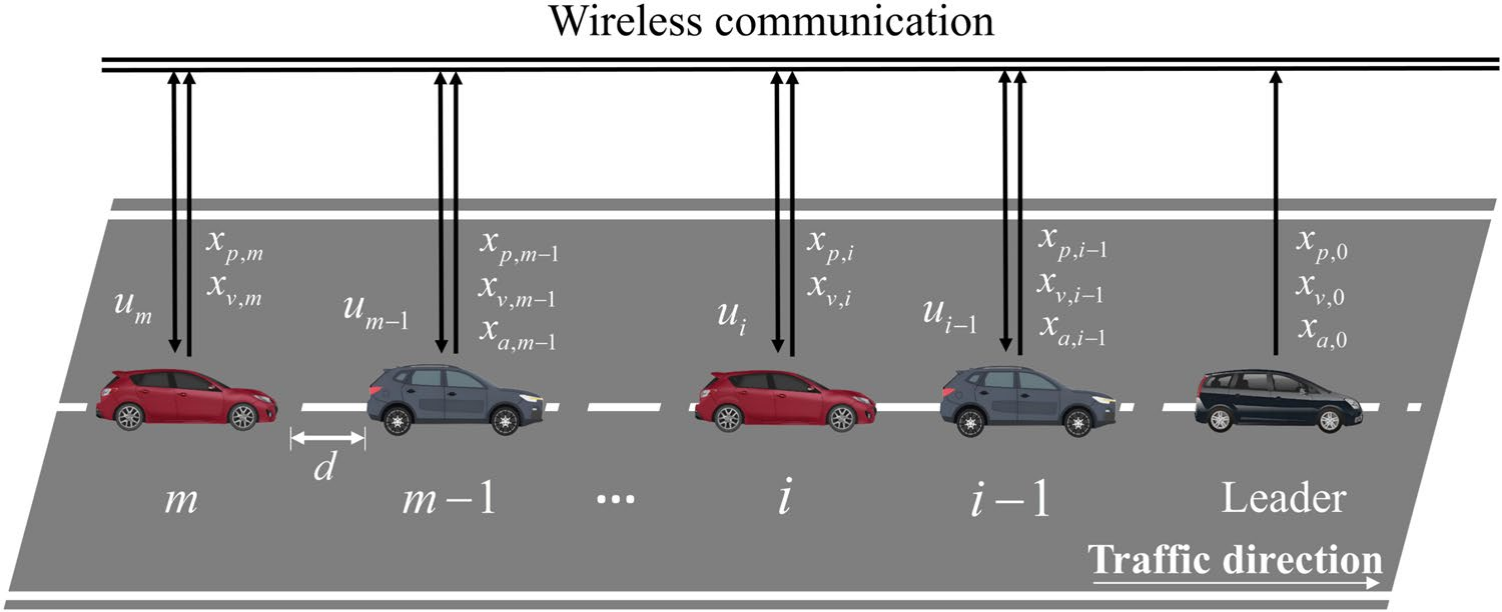
Mixed-order CAVs platoon.

A nonlinear mixed-order CAVs platoon is considered to consist of *M* following vehicles and one leader, as illustrated in Fig. 1. $m_1$ vehicles are second-order and $m_2$ vehicles are third-order. Define $M=\{1,2,\ldots ,m\}$, $M_1=\{1+m_2,2+m_2,\ldots ,m\}$ and $M_2=\{1,2,\ldots ,m_2\}$.

The second-order vehicle dynamics is considered as^35^

1 $$\begin{aligned} \dot{x}_{p,i}(t)&= x_{v,i}(t) \nonumber \\ \dot{x}_{v,i}(t)&= u_i(t) + f_i\left( \bar{\boldsymbol{x}}_i\right) + \epsilon _i, \nonumber \\ f_i\left( \bar{\boldsymbol{x}}_i\right)&=-\frac{\mathcal {R}_i\mathcal {A}_i\mathcal {C}_i}{2\mathcal {M}_i}{x}^2_{v,i}(t) \nonumber \\ \bar{\boldsymbol{x}}_i&=\left[ x_{p,i}(t),x_{v,i}(t),0\right] ^T, i\in M_1 \end{aligned}$$

and the third-order vehicle dynamics is considered as^19^

2 $$\begin{aligned} \dot{x}_{p,i}(t)&= x_{v,i}(t) \nonumber \\ \dot{x}_{v,i}(t)&= x_{a,i}(t) \nonumber \\ \dot{x}_{a,i}(t)&= u_i(t) + f_i\left( \bar{\boldsymbol{x}}_i\right) + \epsilon _i, \nonumber \\ f_i\left( \bar{\boldsymbol{x}}_i\right)&=-\frac{1}{\tau _i}{x}_{a,i}(t) -\frac{\mathcal {R}_i\mathcal {A}_i\mathcal {C}_i}{2\mathcal {M}_i\tau _i}{x}^2_{v,i}(t) \nonumber \\&\quad -\frac{\mathcal {D}_{ri}}{\mathcal {M}_i\tau _i} -\frac{\mathcal {R}_i\mathcal {A}_i\mathcal {C}_i}{\mathcal {M}_i}{x}_{v,i}(t){x}_{a,i}(t) \nonumber \\ \bar{\boldsymbol{x}}_i&=\left[ x_{p,i}(t),x_{v,i}(t),x_{a,i}(t)\right] ^T, i\in M_2 \end{aligned}$$

where $x_{p,i}(t)$, $x_{v,i}(t)$, and $x_{a,i}(t)$ denote the state vectors of position, velocity, and acceleration of the *i*th vehicle, respectively; $f_i\left( \bar{\boldsymbol{x}}_i\right)$ denotes the nonlinear unknown uncertainties and is locally Lipschitz, $\tau _i$ denotes the time lag constant, $\mathcal {R}_i$ is the air mass, $\mathcal {A}_i$ is the cross-sectional area, $\mathcal {C}_i$ denotes the drag coefficient, $\mathcal {D}_{ri}$ denotes the mechanical drag, $\mathcal {M}_i$ denotes the vehicle mass, $u_i$ denotes the control input, $\epsilon _i$ denotes the unknown external disturbances.

### Modeling of actuator saturation and faults

Considering the actuator’s physical limits, the control input is limited to

3 $$\begin{aligned} \begin{aligned} sat(u_i(t)) = {\left\{ \begin{array}{ll} u_{\max }, & u_i(t)\ge u_{\max } \\ u_i(t), & u_{\min }<u_i(t)<\ u_{\max }\\ u_{\min }, & u_i(t)\le u_{\min } \end{array}\right. } \end{aligned} \end{aligned}$$

where $u_{\min }<0$ and $u_{\max }>0$ are the lower and upper bounds of $u_i(t)$.

Rewrite (1) and (2) as

4 $$\begin{aligned} {\left\{ \begin{array}{ll} \dot{x}_{p,i}(t)= x_{v,i}(t) \nonumber \\ \dot{x}_{v,i}(t)= sat(u_i(t)) + f_i\left( \bar{\boldsymbol{x}}_i\right) + \epsilon _i \end{array}\right. }, i\in M_1 \nonumber \\ {\left\{ \begin{array}{ll} \dot{x}_{p,i}(t)= x_{v,i}(t) \\ \dot{x}_{v,i}(t)= x_{a,i}(t) \\ \dot{x}_{a,i}(t)= sat(u_i(t)) + f_i\left( \bar{\boldsymbol{x}}_i\right) + \epsilon _i \end{array}\right. }, i\in M_2 \end{aligned}$$

To reduced the impact of actuator saturation on CAVs platoon, the Gaussian Error Function is employed to approximate $sat(u_i(t))$. Define Gaussian Error Function $\lambda (x )$ as

5 $$\begin{aligned} \lambda (x ) = \frac{2}{{\sqrt{\pi }}}\int _0^{x} {{e^{ - {t^2}}}} dt \end{aligned}$$

By (5), the $sat(u_i(t))$ (3) becomes

6 $$\begin{aligned} \phi \left( u_i(t) \right) = u_{Mi}(t)\lambda \left( \frac{{\sqrt{\pi }u_i(t)}}{{2u_{Mi}(t)}} \right) \end{aligned}$$

where $${u_{Mi}(t)} = \frac{1}{2}({u_{\max }} + {u_{\min }}) + \frac{1}{2}({u_{\max }} - {u_{\min }})sgn({u_i(t)})$$.

Then, we have

7 $$\begin{aligned} sat\left( {{u_i}(t)} \right) = \phi \left( {{u_i}(t)} \right) + {\varepsilon _{u,i}}(t) \end{aligned}$$

where $\varepsilon _{u,i}(t)$ denotes the approximation error of the Gaussian Error Function.

According to the mean-value theorem^36^, $g\left( u_i(t) \right)$ is expressed as

8 $$\begin{aligned} \phi \left( u_i(t) \right) = \phi \left( \overline{u}_i(t) \right) + {\vartheta _i}(t)\left( u_i(t) - \overline{u}_i(t) \right) \end{aligned}$$

where ${\vartheta _i(t)} = {e^{ - {{\left( {\frac{{\sqrt{\pi }\mu _i(t)}}{{2{u_{Mi}(t)}}}} \right) }^2}}}$ and ${\mu _i}(t) = (1 - p)\overline{u}_i(t) + p{u_i}(t)$, with $0< p < 1$.

Let $\overline{u}_i(t) = 0$, one has

9 $$\begin{aligned} sat\left( {{u_i}(t)} \right) = {\vartheta _i}(t){u_i}(t) + {\varepsilon _{u,i}}(t) \end{aligned}$$

where $$0 < {\vartheta _i}(t) \le 1$$.

Then, (4) is rewritten as

10 $$\begin{aligned} {\left\{ \begin{array}{ll} \dot{x}_{p,i}(t)= x_{v,i}(t) \nonumber \\ \dot{x}_{v,i}(t)= {\vartheta _i(t)}{u_i}(t) + {\varepsilon _{u,i}}(t) + f_i\left( \bar{\boldsymbol{x}}_i\right) + \epsilon _i \end{array}\right. }, i\in M_1 \\ {\left\{ \begin{array}{ll} \dot{x}_{p,i}(t)= x_{v,i}(t) \\ \dot{x}_{v,i}(t)= x_{a,i}(t) \\ \dot{x}_{a,i}(t)= {\vartheta _i(t)}{u_i}(t) + {\varepsilon _{u,i}}(t) + f_i\left( \bar{\boldsymbol{x}}_i\right) + \epsilon _i \end{array}\right. }, i\in M_2 \end{aligned}$$

The fault model is considered as

11 $$\begin{aligned} u_{ai}(t)=\rho _i(t)u_i(t)+r_i(t) \end{aligned}$$

where $\rho _i(t)$ is the actuator efficiency factor and $r_i(t)$ is the bias fault, and there exists unknown positive constant $\underline{\rho }_i$ satisfying $1 \ge \rho _i(t) \ge \underline{\rho }_i >0$.

Then, (10) becomes

12 $$\begin{aligned} {\left\{ \begin{array}{ll} \dot{x}_{p,i}(t)= x_{v,i}(t) \\ \dot{x}_{v,i}(t)= {\vartheta _i(t)}\rho _i(t){u_i}(t)+ f_i\left( \bar{\boldsymbol{x}}_i\right) +\varpi _i(t) \end{array}\right. }, i\in M_1 \nonumber \\ {\left\{ \begin{array}{ll} \dot{x}_{p,i}(t)= x_{v,i}(t) \\ \dot{x}_{v,i}(t)= x_{a,i}(t) \\ \dot{x}_{a,i}(t)= {\vartheta _i(t)}\rho _i(t){u_i}(t)+ f_i\left( \bar{\boldsymbol{x}}_i\right) +\varpi _i(t) \end{array}\right. }, i\in M_2 \end{aligned}$$

where $\varpi _i(t)={\varepsilon _{u,i}}(t)+{\vartheta _i}(t) r_i(t)+\epsilon _i$ denotes the lumped disturbances.

Define $$( \bullet )^{M_1}=\begin{bmatrix} {\textbf {0}}_{m-m_1}& & 0\\ 0& & \boldsymbol{I}_{m_1}\end{bmatrix}(\bullet )$$ and $$(\bullet )^{M_2}=\begin{bmatrix}\boldsymbol{I}_{m_2}& 0\\ 0& {\textbf {0}}_{m-m_2}\end{bmatrix}( \bullet )$$. For notational simplicity, we will omit the explicit dependence on time and states. Hence, (12) can be represented globally as

13 $$\begin{aligned} {\left\{ \begin{array}{ll} \dot{\boldsymbol{x}}^{M_1}_{p}= \boldsymbol{x}^{M_1}_{v} \nonumber \\ \dot{\boldsymbol{x}}^{M_1}_{v}= {\boldsymbol{\vartheta }^{M_1}}\boldsymbol{\rho }^{M_1}\boldsymbol{u}^{M_1} + \boldsymbol{f}^{M_1} + \boldsymbol{\varpi }^{M_1} \end{array}\right. } \nonumber \\ {\left\{ \begin{array}{ll} \dot{\boldsymbol{x}}^{M_2}_{p}= \boldsymbol{x}^{M_2}_{v} \\ \dot{\boldsymbol{x}}^{M_2}_{v}= \boldsymbol{x}^{M_2}_{a} \\ \dot{\boldsymbol{x}}^{M_2}_{a}= {\boldsymbol{\vartheta }^{M_2}}\boldsymbol{\rho }^{M_2}\boldsymbol{u}^{M_2} + \boldsymbol{f}^{M_2} + \boldsymbol{\varpi }^{M_2} \end{array}\right. } \end{aligned}$$

where $\boldsymbol{x}_p=\left[ x_{p,1},x_{p,2},\ldots ,x_{p,m}\right] ^T \in \mathbb {R}^{m}$ denotes the position vector, $\boldsymbol{x}_v=\left[ x_{v,1},x_{v,2},\ldots ,x_{v,m}\right] ^T \in \mathbb {R}^{m}$ denotes the velocity vector, $\boldsymbol{x}_a=\left[ x_{a,1},x_{a,2},\ldots ,x_{v,m_2},{\textbf {0}}_{1\times (m-m_2)}\right] ^T \in \mathbb {R}^{m}$ denotes the acceleration vector, $\boldsymbol{\vartheta }=diag\left\{ \vartheta _{1},\vartheta _{2},\ldots ,\vartheta _{m}\right\} \in \mathbb {R}^{m\times m}$, $\boldsymbol{\rho }=diag\{\rho _{1},\rho _{2},\ldots ,\rho _{m}\} \in \mathbb {R}^{m\times m}$, $\boldsymbol{u}=\left[ u_{1},u_{2},\ldots ,u_{m}\right] ^T \in \mathbb {R}^{m}$, $\boldsymbol{f}=\left[ f_{1},f_{2},\ldots ,f_{m}\right] ^T \in \mathbb {R}^{m}$, $\boldsymbol{\varpi }={\boldsymbol{\varepsilon } _{u}}+{\boldsymbol{\vartheta }}\boldsymbol{r}+\boldsymbol{\epsilon }$, $\boldsymbol{\epsilon }=\left[ \epsilon _{1},\epsilon _{2},\ldots ,\epsilon _{m}\right] ^T \in \mathbb {R}^{m}$, $\boldsymbol{\varpi }=\left[ \varpi _{1},\varpi _{2},\ldots ,\varpi _{m}\right] ^T \in \mathbb {R}^{m}$, $\boldsymbol{\varepsilon }_{u}=\left[ \varepsilon _{u,1},\varepsilon _{u,2},\ldots ,\varepsilon _{u,m}\right] ^T \in \mathbb {R}^{m}$, $\boldsymbol{r}=\left[ r_{1},r_{2},\ldots ,r_{m}\right] ^T \in \mathbb {R}^{m}$.

The nonautonomous leader is described by

14 $$\begin{aligned} \dot{x}_{p,0}&= x_{v,0} \nonumber \\ \dot{x}_{v,0}&= x_{a,0} \nonumber \\ \dot{x}_{a,0}&= f_0\left( {\bar{\boldsymbol{x}}_0,t}\right) \end{aligned}$$

where ${x}_{p,0}$, ${x}_{v,0}$, and ${x}_{a,0}$ are the leader’s position, velocity, and acceleration, respectively; $\bar{\boldsymbol{x}}_0=\left[ {x}_{p,0},{x}_{v,0},{x}_{a,0}\right] ^T \in \mathbb {R}^{3}$ represents the leader’s state vector, $f_0\left( {\bar{\boldsymbol{x}}_0,t}\right)$ denotes the unknown nonlinear function.

### Control problem formulation for mixed-order CAVs platoon

The objective of this paper is to design a mixed-order controller for CAVs platoon to achieve the following goals.

1. All following vehicles can track the leader’s velocity, while maintaining the desired inter-vehicle spacing.
2. The neighborhood synchronization errors $e_{p,i}$, $e_{v,i}$, and $e_{a,i}$ are finite-time bounded.

The spacing policy of constant spacing is adopted to ensure that the whole platoon maintains the desired spacing between vehicles. The ideal position for *i*th vehicle is defined as

15 $$\begin{aligned} x_{d,i}=x_{p,0}-i\cdot d,\ i\in M \end{aligned}$$

where $x_{d,i}$ is the *i*th vehicle’s ideal position, *d* represents the standstill distance.

Define the tracking errors as

16 $$\begin{aligned}&{\left\{ \begin{array}{ll} \delta _{p,i}=x_{p,i}-x_{d,i} \\ \delta _{v,i}=x_{v,i}-x_{v,0} \\ \end{array}\right. }, i\in M \nonumber \\&\quad \delta _{a,i}=x_{a,i}-x_{a,0} \quad \ , i \in M_2 \end{aligned}$$

or globally

17 $$\begin{aligned}&{\left\{ \begin{array}{ll} \boldsymbol{\delta }_{p}=\boldsymbol{x}_{p}-\boldsymbol{x}_{d} \\ \boldsymbol{\delta }_{v}=\boldsymbol{x}_{v}-x_{v,0}\boldsymbol{1} \\ \end{array}\right. } \nonumber \\&\quad \boldsymbol{\delta }_{a}=\boldsymbol{x}_{a}-x_{a,0}\boldsymbol{1}^{M_2} \end{aligned}$$

where $\boldsymbol{\delta }_{p}$, $\boldsymbol{\delta }_{v}$, and $\boldsymbol{\delta }_{a}$ represent the position, velocity, and acceleration error, respectively; $\boldsymbol{\delta }_{p}=\left[ \delta _{p,1}, \delta _{p,2},\ldots ,\delta _{p,m}\right] ^T \in \mathbb {R}^{m}$, $\boldsymbol{\delta }_{v}=\left[ \delta _{v,1}, \delta _{v,2},\ldots ,\delta _{v,m}\right] ^T \in \mathbb {R}^{m}$, $\boldsymbol{\delta }_a=\left[ \delta _{a,1},\delta _{a,2},\ldots ,\delta _{a,m_2},{\textbf {0}}_{1\times (m-m_2)}\right] ^T \in \mathbb {R}^{m}$, $\boldsymbol{x}_{d}=[ x_{d,1}, x_{d,2},\ldots ,x_{d,m}] ^T \in \mathbb {R}^{m}$.

Define the neighborhood synchronization errors as

18 $$\begin{aligned} e_{p,i}&=\sum _{j\in \mathcal {N}_{i}}a_{ij}\left( x_{p,j}-x_{p,i}\right) +b_{i}\left( x_{d,i}-x_{p,i}\right) , i\in M \nonumber \\ e_{v,i}&=\sum _{j\in \mathcal {N}_{i}}a_{ij}\left( x_{v,j}-x_{v,i}\right) +b_{i}\left( x_{v,0}-x_{v,i}\right) , i\in M \nonumber \\ e_{a,i}&=\sum _{j\in \mathcal {N}^{*}_{i}}a_{ij}\left( x_{a,j}-x_{a,i}\right) +b_{i}\left( x_{a,0}-x_{a,i}\right) , i\in M_2 \end{aligned}$$

where $e_{p,i}$, $e_{v,i}$, and $e_{a,i}$ represent the position, velocity, and acceleration neighborhood synchronization errors, respectively; $\mathcal {N}_{i}$ represents the neighbors of *i*th following vehicle, $\mathcal {N}^{*}_{i}$ denotes the third-order neighbors of *i*th following vehicle, $b_i \ge 0$ denotes the communication weight between the leader and vehicle *i*, and $b_i > 0$, if and only if there exists a connection from leader to vehicle *i*.

Then, the global synchronization errors can be represented as

19 $$\begin{aligned} \boldsymbol{e}_p&=-(\boldsymbol{L}+\boldsymbol{B})(\boldsymbol{x}_{p}-\boldsymbol{x}_{d})=-(\boldsymbol{L}+\boldsymbol{B})\boldsymbol{\delta }_{p} \nonumber \\ \boldsymbol{e}_v&=-(\boldsymbol{L}+\boldsymbol{B})(\boldsymbol{x}_{v}-x_{v,0}\boldsymbol{1}^{M})=-(\boldsymbol{L}+\boldsymbol{B})\boldsymbol{\delta }_{v} \nonumber \\ \boldsymbol{e}_a&=-(\boldsymbol{L}+\boldsymbol{B})(\boldsymbol{x}_{a}-x_{a,0}\boldsymbol{1}^{M_2})=-(\boldsymbol{L}+\boldsymbol{B})\boldsymbol{\delta }_{a} \end{aligned}$$

where $$\boldsymbol{e}_p=\left[ e_{p,1}, e_{p,2},\ldots ,e_{p,m}\right] ^T \in \mathbb {R}^{m}$$, $$\boldsymbol{e}_v=\left[ e_{v,1}, e_{v,2},\ldots ,e_{v,m}\right] ^T \in \mathbb {R}^{m}$$, $$\boldsymbol{e}_a=\big [ e_{a,1}, e_{a,2},\ldots ,e_{a,m_2},{\textbf {0}}_{1\times (m-m_2)}\big ] ^T \in \mathbb {R}^{m}$$.

#### Remark 1

Define the augmented graph $\mathcal {G}$ as $\bar{\mathcal {G}}=(\bar{\mathcal {V}},\bar{\mathcal {E}})$ with the leader being the root, where $\bar{\mathcal {V}}=\{\mathcal {V}_{0},\ldots ,\mathcal {V}_{m}\}$ and $\bar{{\mathcal {E}}}\subseteq \bar{{\mathcal {V}}}\times \bar{{\mathcal {V}}}$.

#### Assumption 1

(^1^) The augmented graph $\bar{{\mathcal {G}}}$ contains at least one spanning tree for all states with the leader being the root node.

#### Lemma 1

(^1^) Let the pinning matrix $\boldsymbol{B}$ is a positive definite matrix $(\boldsymbol{B}>0)$ and the Laplacian matrix $\boldsymbol{L}$ be irreducible. Then, $\boldsymbol{L} + \boldsymbol{B}$ is nonsingular M-matrix^14^. Define

$$\begin{aligned} \boldsymbol{q}&=(\boldsymbol{L}+\boldsymbol{B})^{-1}\textbf{1}_m=\left[ q_{1},q_{2},\ldots ,q_{m}\right] ^{T} \nonumber \\ \boldsymbol{P}&=diag\left\{ 1/q_{i}\right\} =diag\left\{ p_{i}\right\} , i\in M. \end{aligned}$$

Then, $\boldsymbol{P} > 0$ and define

$$\begin{aligned} \boldsymbol{Q}=\boldsymbol{P}\left( \boldsymbol{L}+\boldsymbol{B}\right) +\left( \boldsymbol{L}+\boldsymbol{B}\right) ^T \boldsymbol{P}. \end{aligned}$$

Then, $$\boldsymbol{Q} > 0$$.

#### Lemma 2

(^14^) Let the pinning matrix $\boldsymbol{B}\ne 0$ and the augmented graph $\bar{\boldsymbol{\mathcal {G}}}$ contains at least one spanning tree. Then

$$\begin{aligned} \left\| \boldsymbol{\delta }_l\right\| \le \left\| \boldsymbol{e}_l\right\| /\underline{\sigma }\left( \boldsymbol{L}+\boldsymbol{B}\right) , \ l=p,v,a \end{aligned}$$

As $\boldsymbol{L} + \boldsymbol{B}$ is nonsingular, based on (17) and (19), $\boldsymbol{\delta }_{l}=-(\boldsymbol{L}+\boldsymbol{B})^{-1}\boldsymbol{e}_l$. That is $\boldsymbol{e}_l=-(\boldsymbol{L}+\boldsymbol{B})\boldsymbol{\delta }_{l}$. Then, we can obtain $\Vert \boldsymbol{\delta }_{l}\Vert =\Vert (\boldsymbol{L}+\boldsymbol{B})^{-1}\boldsymbol{e}_l\Vert \le \left\| \boldsymbol{e}_l\right\| /\underline{\sigma }\left( \boldsymbol{L}+\boldsymbol{B}\right)$ with $\boldsymbol{e}_l = 0$, if and only if all the vehicles can achieve the synchronization.

#### Definition 1

(^37^) $\boldsymbol{\delta }_l$ is cooperatively uniformly ultimately bounded (CUUB) if there exists a compact set $\Omega _{l}\subset R^{m}$, so that for $\forall \boldsymbol{\delta }_l(t_{0}) \in \Omega _{l}$ there exist bound $B_l$ and time $T_l\left[ B_l, \boldsymbol{\delta }_p(t_{0}), \boldsymbol{\delta }_v(t_{0}), \boldsymbol{\delta }_a(t_{0})\right]$, such that $\left\| \boldsymbol{\delta }_l(t)\right\| \le B_{l}$, $\forall t \ge t_0 + T_l$.

Thus, if $\boldsymbol{\delta }_p$, $\boldsymbol{\delta }_v$, and $\boldsymbol{\delta }_a$ are CUUB, and $x_{p,i}$, $x_{v,i}$ and $x_{a,i}$ are bounded, all following vehicles can achieve state synchronization to the leader.

#### Lemma 3

[^38^] There exists a continuous function $\mathbb {V}(t)\ge 0$ with $\mathbb {V}(0)$ is bounded. If $\mathbb {V}(t)$ satisfies $\dot{\mathbb {V}}(t)\le -\mathbb {B}_1 \mathbb {V}(t)+\mathbb {B}_2$, where $\mathbb {B}_1>0$ and $\mathbb {B}_2$ being a constant, then, $\mathbb {V}(t)$ is bounded.

#### Lemma 4

(Young’s inequality^39^) For vectors $\boldsymbol{\wp }$, $\boldsymbol{\Im } \in \mathbb {R}^m$ and $\hbar > 0$, it holds that

$$\begin{aligned} \boldsymbol{\wp }^T \boldsymbol{\Im } \le \frac{\hbar }{2} \boldsymbol{\wp }^T \boldsymbol{\wp } + \frac{1}{2\hbar } \boldsymbol{\Im }^T \boldsymbol{\Im }. \end{aligned}$$

### NNs approximation

As NNs are capable of approximating any smooth nonlinear function and possess strong self-learning abilities, they are utilized to approximate uncertainties and actuator efficiency factor. Following the techniques mentioned in^2^, unknown continuous nonlinear function ${f}(\boldsymbol{Z})$ defined on $\Omega \in {\mathbb {R}^{N}}$ is approximated by RBFNN as

20 $$\begin{aligned} {f}(\boldsymbol{Z}) = \boldsymbol{W}^T{\boldsymbol{h}}(\boldsymbol{Z}) + {\varepsilon } \end{aligned}$$

where $\boldsymbol{Z}=\left[ z_1, z_2, \ldots , z_N\right] ^T \in \mathbb {R}^{N}$ represents the input vector, ${\boldsymbol{W}} = \left[ {{{W}_1},{{W}_2}, \ldots ,{{W}_{N_s}}} \right] ^T \in \mathbb {R}^{N_s}$ represents the weight vector with ${N_s}$ being the number of neurons, ${\varepsilon }$ denotes the approximation error, and ${\boldsymbol{h}}\left( \boldsymbol{Z} \right) = {\left[ {{{h}_1 }(\boldsymbol{Z}),{{h}_2}(\boldsymbol{Z}), \ldots ,{{h}_{N_s}}(\boldsymbol{Z})} \right] ^T} \in \mathbb {R}^{N_s}$ denotes the Gaussian basis function vector with ${ h_k}\left( \boldsymbol{Z} \right)$ is

21 $$\begin{aligned} {{h}_k}\left( \boldsymbol{Z} \right) = \exp \left[ { - \frac{{{{\left( {\boldsymbol{Z} - {\boldsymbol{y}_k}} \right) }^T} \left( {\boldsymbol{Z} - {\boldsymbol{y}_k}} \right) }}{{c_k^2}}} \right] , k = 1,2, \ldots ,{N_s} \end{aligned}$$

where ${\boldsymbol{y}_k} = {\left[ {{y_{k,1}},{y_{k,2}}, \ldots ,{y_{k,{N_s}}}} \right] ^{T}} \in \mathbb {R}^{N_s}$ represents the center of the receptive field, $c_k$ denotes the width value.

According to (20), ${f}_i\left( \bar{\boldsymbol{x}}_i\right)$ and $\rho _i\left( \bar{\boldsymbol{x}}_i,t\right)$ can be approximated as

22 $$\begin{aligned} {\left\{ \begin{array}{ll} {f}_i\left( \bar{\boldsymbol{x}}_i\right) = {\boldsymbol{\omega }^{T}_{f,i}}{\boldsymbol{h}_{f,i}}\left( \bar{\boldsymbol{x}}_i\right) + {\varepsilon _{f,i}} \\ \rho _i\left( \bar{\boldsymbol{x}}_i,t\right) = {\boldsymbol{\omega }^{T}_{\rho ,i}}{\boldsymbol{h}_{\rho ,i}}\left( \bar{\boldsymbol{x}}_i\right) + {\varepsilon _{\rho ,i}} \end{array}\right. }, \ i\in M \end{aligned}$$

where ${\boldsymbol{\omega }_{f,i}} \in \mathbb {R}^{N_s}$ and $\boldsymbol{\omega }_{\rho ,i} \in \mathbb {R}^{N_s}$ denote the ideal weight vector, ${\boldsymbol{h}_{f,i}} \in \mathbb {R}^{N_s}$ and ${\boldsymbol{h}_{\rho ,i}} \in \mathbb {R}^{N_s}$ are the basis function, ${\varepsilon _{f,i}}$ and ${\varepsilon _{\rho ,i}}$ are the RBF approximation error.

Define $\hat{f}_i\left( \bar{\boldsymbol{x}}_i\right)$ and ${\hat{\rho }}_i\left( \bar{\boldsymbol{x}}_i,t\right)$ as

23 $$\begin{aligned} {\left\{ \begin{array}{ll} \hat{f}_i\left( \bar{\boldsymbol{x}}_i\right) = {\hat{\boldsymbol{\omega }}^{T}_{f,i}}{\boldsymbol{h}_{f,i}}\left( \bar{\boldsymbol{x}}_i\right) \\ {\hat{\rho }}_i\left( \bar{\boldsymbol{x}}_i,t\right) = {\hat{\boldsymbol{\omega }}^{T}_{\rho ,i}}{\boldsymbol{h}_{\rho ,i}}\left( \bar{\boldsymbol{x}}_i\right) \end{array}\right. },\ i\in M \end{aligned}$$

where $\hat{f}_i\left( \bar{\boldsymbol{x}}_i\right)$ and $\hat{\rho }_i\left( \bar{\boldsymbol{x}}_i,t\right)$ are the estimations of ${f}_i\left( \bar{\boldsymbol{x}}_i\right)$ and $\rho _i\left( \bar{\boldsymbol{x}}_i,t\right)$, ${\hat{\boldsymbol{\omega }}_{f,i}}$ and ${\hat{\boldsymbol{\omega }}_{\rho ,i}}$ are the estimations of ${\boldsymbol{\omega }_{f,i}}$ and ${\boldsymbol{\omega }_{\rho ,i}}$, respectively.

Then, (22) can be represented globally as

24 $$\begin{aligned} {\left\{ \begin{array}{ll} \boldsymbol{f}(\bar{\boldsymbol{x}}) = {\boldsymbol{\omega }^{T}_{f}}{\boldsymbol{h}_{f}}\left( \bar{\boldsymbol{x}}\right) + {\boldsymbol{\varepsilon } _{f}} \\ \boldsymbol{\rho }\left( \bar{\boldsymbol{x}},t\right) = {\boldsymbol{\omega }^{T}_{\rho }}{\boldsymbol{h}_{\rho }}\left( \bar{\boldsymbol{x}}\right) + {\boldsymbol{\varepsilon } _{\rho }} \end{array}\right. } \end{aligned}$$

where $${\boldsymbol{\omega }_f}=diag\left\{ \boldsymbol{\omega }_{f,1}, \boldsymbol{\omega }_{f,2}, \ldots , \boldsymbol{\omega }_{f,m}\right\} \in \mathbb {R}^{m\cdot N_s\times m}$$, $${\boldsymbol{h}_f}\left( \bar{\boldsymbol{x}}\right) =\left[ {\boldsymbol{h}^T_{f,1}}\left( \bar{\boldsymbol{x}}_1\right) , {\boldsymbol{h}^T_{f,2}}\left( \bar{\boldsymbol{x}}_2\right) , \ldots , {\boldsymbol{h}^T_{f,m}}\left( \bar{\boldsymbol{x}}_m\right) \right] ^T \in \mathbb {R}^{m\cdot N_s}$$, $${\boldsymbol{\varepsilon }_{f}}=[ \varepsilon _{f,1}, \varepsilon _{f,2},\ldots , \varepsilon _{f,m}] ^T \in \mathbb {R}^{m}$$, $${\boldsymbol{\omega }_\rho }=diag\left\{ \boldsymbol{\omega }_{\rho ,1}, \boldsymbol{\omega }_{\rho ,2}, \ldots , \boldsymbol{\omega }_{\rho ,m}\right\} \in \mathbb {R}^{m \cdot N_s\times m}$$, $${\boldsymbol{h}_\rho }\left( \bar{\boldsymbol{x}}\right) =diag \left\{ {\boldsymbol{h}^T_{\rho ,1}}\left( \bar{\boldsymbol{x}}_1\right) , {\boldsymbol{h}^T_{\rho ,2}}\left( \bar{\boldsymbol{x}}_2\right) , \ldots , {\boldsymbol{h}^T_{\rho ,m}}\left( \bar{\boldsymbol{x}}_m\right) \right\} \in \mathbb {R}^{m\cdot N_s\times m}$$, $${\boldsymbol{\varepsilon }_{\rho }}=\left[ \varepsilon _{\rho ,1}, \varepsilon _{\rho ,2}, \ldots , \varepsilon _{\rho ,m}\right] ^T \in \mathbb {R}^{m}$$.

Then, (23) can be represented globally as

25 $$\begin{aligned} {\left\{ \begin{array}{ll} \hat{\boldsymbol{f}}(\bar{\boldsymbol{x}}) = {\hat{\boldsymbol{\omega }}^{T}_{f}}{\boldsymbol{h}_{f}}\left( \bar{\boldsymbol{x}}\right) \\ \hat{\boldsymbol{\rho }}\left( \bar{\boldsymbol{x}},t\right) = {\hat{\boldsymbol{\omega }}^{T}_{\rho }}{\boldsymbol{h}_{\rho }}\left( \bar{\boldsymbol{x}}\right) \end{array}\right. }\ \end{aligned}$$

where $$\hat{\boldsymbol{f}}\left( \bar{\boldsymbol{x}}\right) =[ \hat{f}_1\left( \bar{\boldsymbol{x}}_1\right) , \hat{f}_2\left( \bar{\boldsymbol{x}}_2\right) , \ldots ,\hat{f}_m\left( \bar{\boldsymbol{x}}_m\right) ] ^T \in \mathbb {R}^{m}$$, $${\hat{\boldsymbol{\omega }}_{f}}=diag\left\{ {\hat{\boldsymbol{\omega }}_{f,1}}, {\hat{\boldsymbol{\omega }}_{f,2}}, \ldots ,{\hat{\boldsymbol{\omega }}_{f,m}}\right\} \in \mathbb {R}^{m\cdot N_s\times m}$$, $$\hat{\boldsymbol{\rho }}\left( \bar{\boldsymbol{x}},t\right) =diag\{ \hat{{\rho }}_1(\bar{\boldsymbol{x}}_1,t), \hat{{\rho }}_2(\bar{\boldsymbol{x}}_2,t), \ldots ,\hat{{\rho }}_m(\bar{\boldsymbol{x}}_m,t)\}\in \mathbb {R}^{m\times m}$$, $${\hat{\boldsymbol{\omega }}_{\rho }}=diag\left\{ {\hat{\boldsymbol{\omega }}_{\rho ,1}}, {\hat{\boldsymbol{\omega }}_{\rho ,2}}, \ldots ,{\hat{\boldsymbol{\omega }}_{\rho ,m}}\right\} \in \mathbb {R}^{m\cdot N_s\times m}$$.

#### Remark 2

Define $h_{f,iM}=\max \limits _{\bar{\boldsymbol{x}}_i\in \Omega }\Vert \boldsymbol{h}_{f,i}(\bar{\boldsymbol{x}}_i)\Vert$, $h_{\rho ,iM}=\max \limits _{\bar{\boldsymbol{x}}_i\in \Omega }\Vert \boldsymbol{h}_{\rho ,i}(\bar{\boldsymbol{x}}_i)\Vert$, ${\omega _{f,iM}}=\left\| \boldsymbol{\omega }_{f,i}\right\| _F$, and ${\omega _{\rho ,iM}}=\left\| \boldsymbol{\omega }_{\rho ,i}\right\| _F$, where $h_{f,iM}$,$h_{\rho ,iM}$,${\omega _{f,iM}}$,${\omega _{\rho ,iM}}$ are positive constants. Then, there exist positive $h_{fM}$, $h_{\rho M}$, ${\omega _{fM}}$, and ${\omega _{\rho M}}$, such that $\left\| {\boldsymbol{h}_{f}(\bar{\boldsymbol{x}})}\right\| _F \le h_{fM}$, $\left\| {\boldsymbol{h}_{\rho }(\bar{\boldsymbol{x}})}\right\| _F \le h_{\rho M}$, $\left\| {\boldsymbol{\omega }_{f}}\right\| _F \le \omega _{f M}$, and $\left\| {\boldsymbol{\omega }_{\rho }}\right\| _F=\omega _{\rho M}$. $\varepsilon _{f}$ and $\varepsilon _{\rho }$ are bounded by $\left\| \boldsymbol{\varepsilon }_{f}\right\| \le \varepsilon _{fM}$ and $\left\| \boldsymbol{\varepsilon }_{\rho }\right\| \le \varepsilon _{\rho M}$ with constants $\varepsilon _{fM} > 0$, $\varepsilon _{\rho M} > 0$.

#### Remark 3

The RBFNN approximation technique is employed due to its universal approximation capability, simple three-layer structure with linear output weights, localized activation property of Gaussian basis functions. Compared to traditional adaptive control, RBFNN offer superior dynamic performance with lower computational overhead due to their localized learning mechanism, making them particularly suitable for real-time online adaptation in this study.

## Distributed adaptive fault-tolerant control design and stability analysis

### ADOB Design

This part focuses on designing a novel ADOB to estimate and compensate for lumped disturbances, which can ensure the system stability and improve control performance of mixed-order CAVs platoon.

#### Assumption 2

(^40^) There exists unknown constant $\xi$ such that global $\boldsymbol{\varpi }$ is bounded by $\Vert \boldsymbol{\varpi }\Vert \le \xi$.

Define the dynamic error of ADOB as

26 $$\begin{aligned} \mathcal {S}_i(t)= {\left\{ \begin{array}{ll} x_{v,i}(t)-{\hat{x}}_{v,i}(t), & i\in M_1 \\ x_{a,i}(t)-{\hat{x}}_{a,i}(t), & i\in M_2 \\ \end{array}\right. } \end{aligned}$$

where ${\hat{x}}_{v,i}(t)$ and ${\hat{x}}_{a,i}(t)$ are the estimations of $x_{v,i}(t)$ and $x_{a,i}(t)$, respectively.

Globally, (26) can be expressed as

27 $$\begin{aligned} \boldsymbol{\mathcal {S}}&=\boldsymbol{\mathcal {S}}_{m_1}+\boldsymbol{\mathcal {S}}_{m_2} \nonumber \\ \boldsymbol{\mathcal {S}}_{m_1}&=\boldsymbol{x}^{M_1}_v - \hat{\boldsymbol{x}}^{M_1}_v \nonumber \\ \boldsymbol{\mathcal {S}}_{m_2}&=\boldsymbol{x}^{M_2}_a - \hat{\boldsymbol{x}}^{M_2}_a \end{aligned}$$

where $$\hat{\boldsymbol{x}}_v=\left[ {\hat{x}}_{v,1},{\hat{x}}_{v,2},\ldots ,{\hat{x}}_{v,m}\right] ^T \in \mathbb {R}^{m}$$, $$\hat{\boldsymbol{x}}_a=\left[ {\hat{x}}_{a,1},{\hat{x}}_{a,2},\ldots ,{\hat{x}}_{a,m_2},{\textbf {0}}_{1\times (m-m_2)}\right] ^T \in \mathbb {R}^{m}$$.

Design the ADOB as

28 $$\begin{aligned} {\left\{ \begin{array}{ll} \dot{{\hat{x}}}_{v,i}(t) = {\vartheta _i}\rho _i(t){u_i}(t) +f_i\left( \bar{\boldsymbol{x}}_i\right) +{\hat{\varpi }}_i(t) \\ \hspace{0.8mm} {\hat{\varpi }}_i(t) \hspace{0.5mm} = \alpha _{s} \mathcal {S}_i(t)+{\hat{\xi }}_i(t)\\ \hspace{1mm}\dot{{\hat{\xi }}}_i(t) \hspace{1.5mm} = {\aleph }\mathcal {S}_i(t)-{\aleph }\alpha _{\xi }{\hat{\xi }}_i(t) \end{array}\right. },i\in M_1 \nonumber \\ {\left\{ \begin{array}{ll} \dot{{\hat{x}}}_{a,i}(t) = {\vartheta _i}\rho _i(t){u_i}(t) +f_i\left( \bar{\boldsymbol{x}}_i\right) +{\hat{\varpi }}_i(t) \\ \hspace{0.8mm}{\hat{\varpi }}_i(t) \hspace{0.5mm} = \alpha _{s} \mathcal {S}_i(t)+{\hat{\xi }}_i(t)\\ \hspace{1mm} \dot{{\hat{\xi }}}_i(t) \hspace{1.5mm} = {\aleph }\mathcal {S}_i(t)-{\aleph }\alpha _{\xi }{\hat{\xi }}_i(t) \end{array}\right. },i\in M_2 \end{aligned}$$

where $\hat{\varpi }_i(t)$ and ${\hat{\xi }}_i(t)$ are the estimations of ${\varpi }_i(t)$ and $\xi _i(t)$, respectively; ${\aleph }$, $\alpha _{s}$, and $\alpha _{\xi }$ are positive constants.

Globally, (28) can be expressed as

29 $$\begin{aligned} {\left\{ \begin{array}{ll} \hspace{1mm}\dot{\hat{\boldsymbol{x}}}^{M_1}_{v}= {\boldsymbol{\vartheta }^{M_1}}\boldsymbol{\rho }^{M_1}\boldsymbol{u}^{M_1} + \boldsymbol{f}^{M_1} + \hat{\boldsymbol{\varpi }}^{M_1} \\ \hat{\boldsymbol{\varpi }}^{M_1} = \alpha _{s} \boldsymbol{\mathcal {S}}_{m_1} + \hat{\boldsymbol{\xi }}^{M_1} \\ \hspace{1.2mm}\dot{\hat{\boldsymbol{\xi }}}^{M_1} \hspace{0.4mm} = {\aleph }\boldsymbol{\mathcal {S}}_{m_1} - {\aleph }\alpha _{\xi }\hat{\boldsymbol{\xi }}^{M_1} \end{array}\right. } \nonumber \\ {\left\{ \begin{array}{ll} \hspace{1mm} \dot{\hat{\boldsymbol{x}}}^{M_2}_{a}= {\boldsymbol{\vartheta }^{M_2}}\boldsymbol{\rho }^{M_2}\boldsymbol{u}^{M_2} + \boldsymbol{f}^{M_2} + \hat{\boldsymbol{\varpi }}^{M_2} \\ \hat{\boldsymbol{\varpi }}^{M_2} = \alpha _{s} \boldsymbol{\mathcal {S}}_{m_2} + \hat{\boldsymbol{\xi }}^{M_2} \\ \hspace{1mm} \dot{\hat{\boldsymbol{\xi }}}^{M_2} \hspace{0.4mm} = {\aleph }\boldsymbol{\mathcal {S}}_{m_2} - {\aleph }\alpha _{\xi }\hat{\boldsymbol{\xi }}^{M_2} \end{array}\right. } \end{aligned}$$

where $$\hat{\boldsymbol{\varpi }}=\left[ {\hat{\varpi }}_{1},\hat{\varpi }_{2},\ldots ,{\hat{\varpi }}_{m}\right] ^T \in \mathbb {R}^{m}$$, $$\hat{\boldsymbol{\xi }} =\left[ {\hat{\xi }}_{1},{\hat{\xi }}_{2},\ldots ,\hat{\xi }_{m}\right] ^T \in \mathbb {R}^{m}$$.

#### Theorem 1

For the mixed-order CAVs (13) with the lumped disturbances under Assumption 2, the proposed ADOB (28) can make the estimated disturbance errors $\tilde{\varpi }_i(t)=\varpi _i(t)-\hat{\varpi }_i(t)$ converge to a bounded set.

#### Proof

Define $( \tilde{\bullet }) = ( \bullet ) - ( {\hat{\bullet }} )$. Based on (29), differentiating (27), we have

30 $$\begin{aligned} \dot{\boldsymbol{\mathcal {S}}}&=\dot{\boldsymbol{\mathcal {S}}}_{m_1}+\dot{\boldsymbol{\mathcal {S}}}_{m_2} \nonumber \\&={\boldsymbol{\boldsymbol{\vartheta }}^{M_1}}\boldsymbol{\rho }^{M_1}\boldsymbol{u}^{M_1} + \boldsymbol{f}^{M_1} + \boldsymbol{\varpi }^{M_1}-\left( {\boldsymbol{\vartheta }^{M_1}}\boldsymbol{\rho }^{M_1}\boldsymbol{u}^{M_1} + \boldsymbol{f}^{M_1} + \hat{\boldsymbol{\varpi }}^{M_1}\right) \nonumber \\&\quad + {\boldsymbol{\vartheta }^{M_2}}\boldsymbol{\rho }^{M_2}\boldsymbol{u}^{M_2} + \boldsymbol{f}^{M_2} + \boldsymbol{\varpi }^{M_2} - \left( {\boldsymbol{\vartheta }^{M_2}}\boldsymbol{\rho }^{M_2}\boldsymbol{u}^{M_2} + \boldsymbol{f}^{M_2} + \hat{\boldsymbol{\varpi }}^{M_2}\right) \nonumber \\&=\boldsymbol{\varpi }^{M_1} -\hat{\boldsymbol{\varpi }}^{M_1} + \boldsymbol{\varpi }^{M_2} -\hat{\boldsymbol{\varpi }}^{M_2} = \tilde{\boldsymbol{\varpi }}^{M_1}+\tilde{\boldsymbol{\varpi }}^{M_2} \end{aligned}$$

Define the Lyapunov function as

31 $$\begin{aligned} V_D&=\frac{1}{2}\boldsymbol{\mathcal {S}}^{T}\boldsymbol{\mathcal {S}} + \frac{1}{2\aleph }tr\left\{ (\tilde{\boldsymbol{\xi }}^M)^T \tilde{\boldsymbol{\xi }}^M\right\} \end{aligned}$$

The differential of $V_D$ is expressed as

32 $$\begin{aligned} \dot{V}_D&=\boldsymbol{\mathcal {S}}^T\dot{\boldsymbol{\mathcal {S}}} + \frac{1}{\aleph }tr\left\{ (\tilde{\boldsymbol{\xi }}^M)^T \dot{\tilde{\boldsymbol{\xi }}}^M\right\} \nonumber \\&=\boldsymbol{\mathcal {S}}^T(\boldsymbol{\varpi }^{M_1} -\hat{\boldsymbol{\varpi }}^{M_1} + {\boldsymbol{\varpi }}^{M_2} -\hat{\boldsymbol{\varpi }}^{M_2}) - \frac{1}{\aleph }tr\left\{ (\tilde{\boldsymbol{\xi }}^{M_1})^T \dot{\hat{\boldsymbol{\xi }}}^{M_1}\right\} - \frac{1}{\aleph }tr\left\{ (\tilde{\boldsymbol{\xi }}^{M_2})^T \dot{\hat{\boldsymbol{\xi }}}^{M_2}\right\} \nonumber \\&=\boldsymbol{\mathcal {S}}^T_{m_1} {\boldsymbol{\varpi }}^{M_1}-\alpha _{s}\boldsymbol{\mathcal {S}}^T_{m_1} \boldsymbol{\mathcal {S}}_{m_1} - \boldsymbol{\mathcal {S}}^T_{m_1} \hat{\boldsymbol{\xi }}^{M_1} +\boldsymbol{\mathcal {S}}^T_{m_2} {\boldsymbol{\varpi }}^{M_2}-\alpha _{s}\boldsymbol{\mathcal {S}}^T_{m_2} \boldsymbol{\mathcal {S}}_{m_2} - \boldsymbol{\mathcal {S}}^T_{m_2} \hat{\boldsymbol{\xi }}^{M_2}\nonumber \\&\quad - \frac{1}{\aleph }tr\left\{ (\tilde{\boldsymbol{\xi }}^{M_1})^T \dot{\hat{\boldsymbol{\xi }}}^{M_1}\right\} - \frac{1}{\aleph }tr\left\{ (\tilde{\boldsymbol{\xi }}^{M_2})^T \dot{\hat{\boldsymbol{\xi }}}^{M_2}\right\} \end{aligned}$$

Based on $\boldsymbol{\mathcal {S}}^T \boldsymbol{\varpi } \le \boldsymbol{\mathcal {S}}^T\Vert \boldsymbol{\varpi }\Vert$ and Assumption 2, we have

33 $$\begin{aligned} \dot{V}_D&\le \boldsymbol{\mathcal {S}}^T_{m_1} \xi ^{M_1}-\alpha _{s}\boldsymbol{\mathcal {S}}^T_{m_1} \boldsymbol{\mathcal {S}}_{m_1} - \boldsymbol{\mathcal {S}}^T_{m_1} \hat{\boldsymbol{\xi }}^{M_1} +\boldsymbol{\mathcal {S}}^T_{m_2} \xi ^{M_2}-\alpha _{s}\boldsymbol{\mathcal {S}}^T_{m_2} \boldsymbol{\mathcal {S}}_{m_2} - \boldsymbol{\mathcal {S}}^T_{m_2} \hat{\boldsymbol{\xi }}^{M_2} \nonumber \\&\quad - \frac{1}{\aleph }tr\left\{ (\tilde{\boldsymbol{\xi }}^{M_1})^T \dot{\hat{\boldsymbol{\xi }}}^{M_1}\right\} - \frac{1}{\aleph }tr\left\{ (\tilde{\boldsymbol{\xi }}^{M_2})^T \dot{\hat{\boldsymbol{\xi }}}^{M_2}\right\} \nonumber \\&\le \boldsymbol{\mathcal {S}}^T_{m_1} \tilde{\boldsymbol{\xi }}^{M_1} -\alpha _{s}\boldsymbol{\mathcal {S}}^T_{m_1} \boldsymbol{\mathcal {S}}_{m_1} - \frac{1}{\aleph }tr\left\{ (\tilde{\boldsymbol{\xi }}^{M_1})^T \dot{\hat{\boldsymbol{\xi }}}^{M_1}\right\} + \boldsymbol{\mathcal {S}}^T_{m_2} \tilde{\boldsymbol{\xi }}^{M_2} -\alpha _{s} \boldsymbol{\mathcal {S}}^T_{m_2} \boldsymbol{\mathcal {S}}_{m_2} - \frac{1}{\aleph }tr\left\{ (\tilde{\boldsymbol{\xi }}^{M_2})^T \dot{\hat{\boldsymbol{\xi }}}^{M_2}\right\} \end{aligned}$$

Considering $\boldsymbol{x}^T\boldsymbol{y}={tr}\{\boldsymbol{y}\boldsymbol{x}^T\}$ and (29), we have

34 $$\begin{aligned} \dot{V}_D&\le tr\left\{ \tilde{\boldsymbol{\xi }}^{M_1} \boldsymbol{\mathcal {S}}^T_{m_1} \right\} -\alpha _{s} \boldsymbol{\mathcal {S}}^T_{m_1} \boldsymbol{\mathcal {S}}_{m_1} - \frac{1}{\aleph }tr\left\{ (\tilde{\boldsymbol{\xi }}^{M_1})^T \dot{\hat{\boldsymbol{\xi }}}^{M_1}\right\} \nonumber \\&\quad + tr\left\{ \tilde{\boldsymbol{\xi }}^{M_2} \boldsymbol{\mathcal {S}}^T_{m_2} \right\} -\alpha _{s}\boldsymbol{\mathcal {S}}^T_{m_2} \boldsymbol{\mathcal {S}}_{m_2} - \frac{1}{\aleph }tr\left\{ (\tilde{\boldsymbol{\xi }}^{M_2})^T \dot{\hat{\boldsymbol{\xi }}}^{M_2}\right\} \nonumber \\&\le tr\left\{ (\tilde{\boldsymbol{\xi }}^{M_1})^T (\boldsymbol{\mathcal {S}}_{m_1} - \frac{1}{\aleph }\dot{\hat{\boldsymbol{\xi }}}^{M_1}) \right\} -\alpha _{s}\boldsymbol{\mathcal {S}}^T_{m_1} \boldsymbol{\mathcal {S}}_{m_1} + tr\left\{ (\tilde{\boldsymbol{\xi }}^{M_2})^T (\boldsymbol{\mathcal {S}}_{m_2} - \frac{1}{\aleph }\dot{\hat{\boldsymbol{\xi }}}^{M_2}) \right\} -\alpha _{s} \boldsymbol{\mathcal {S}}^T_{m_2} \boldsymbol{\mathcal {S}}_{m_2} \nonumber \\&\le \alpha _{\xi } tr\left\{ ( \tilde{\boldsymbol{\xi }}^{M_1})^T {\hat{\boldsymbol{\xi }}}^{M_1} \right\} -\alpha _{s}\boldsymbol{\mathcal {S}}^T_{m_1} \boldsymbol{\mathcal {S}}_{m_1} + \alpha _{\xi }tr\left\{ (\tilde{\boldsymbol{\xi }}^{M_2})^T {\hat{\boldsymbol{\xi }}}^{M_2} \right\} -\alpha _{s} \boldsymbol{\mathcal {S}}^T_{m_2} \boldsymbol{\mathcal {S}}_{m_2} \end{aligned}$$

By Lemma 4, we have

35 $$\begin{aligned} \dot{V}_D&\le -{\alpha _s}\boldsymbol{\mathcal {S}}^T_{m_1} \boldsymbol{\mathcal {S}}_{m_1} -{\alpha _s}\boldsymbol{\mathcal {S}}^T_{m_2} \boldsymbol{\mathcal {S}}_{m_2} - \frac{{\alpha _\xi }}{2} tr\{(\tilde{\boldsymbol{\xi }}^{M_1})^T {\tilde{\boldsymbol{\xi }}}^{M_1} \} +\frac{{\alpha _\xi }}{2} tr\{({\boldsymbol{\xi }}^{M_1})^T {\boldsymbol{\xi }}^{M_1} \} \nonumber \\&\quad - \frac{{\alpha _\xi }}{2} tr\{(\tilde{\boldsymbol{\xi }}^{M_2})^T {\tilde{\boldsymbol{\xi }}}^{M_2} \} +\frac{{\alpha _\xi }}{2} tr\{({\boldsymbol{\xi }}^{M_2})^T {\boldsymbol{\xi }}^{M_2} \} \nonumber \\&\le -\Pi _{1}{V}_D + \Pi _{2} \end{aligned}$$

where $\Pi _{1} =\frac{\min \left\{ 2{\alpha _{s}},{\alpha _{\xi }}\right\} }{\max \left\{ 1,\aleph \right\} }$, $\Pi _{2} = \frac{{\alpha _\xi }}{2} tr\{(\boldsymbol{\xi }^{M})^T {\boldsymbol{\xi }}^{M} \}$. By Lemma 3, ${V}_D$ is bounded.

Then, Based on Corollary 1.1 in^41^, we have

36 $$\begin{aligned} {V}_D \le \left[ {{V}_D(0) - \frac{\Pi _{2}}{\Pi _{1}}} \right] {e^{ - \Pi _{1}t}} + \frac{\Pi _{2}}{\Pi _{1}} \le {V}_D(0) + \frac{\Pi _{2}}{\Pi _{1}} \end{aligned}$$

where ${V}_D(0)$ is the initial value of ${V}_D$.

Then, one can obtain

37 $$\begin{aligned} \lim _{t\rightarrow \infty }\frac{1}{2}\boldsymbol{\mathcal {S}}^{T}\boldsymbol{\mathcal {S}} \le {V}_D(0) + \frac{\Pi _{2}}{\Pi _{1}} \end{aligned}$$

Further, we have

38 $$\begin{aligned} \Vert \boldsymbol{\mathcal {S}}\Vert \le \sqrt{2\left( {V}_D(0) + \frac{\Pi _{2}}{\Pi _{1}}\right) } \end{aligned}$$

According to (38), $\boldsymbol{\mathcal {S}}$ can converge to a bounded neighborhood of zero. Therefore, the proposed ADOB can ensure that $\tilde{\varpi }_i(t)$ converge to a bounded set. The proof of Theorem 1 is completed. $\square$

### Controller design

**Fig. 2:**
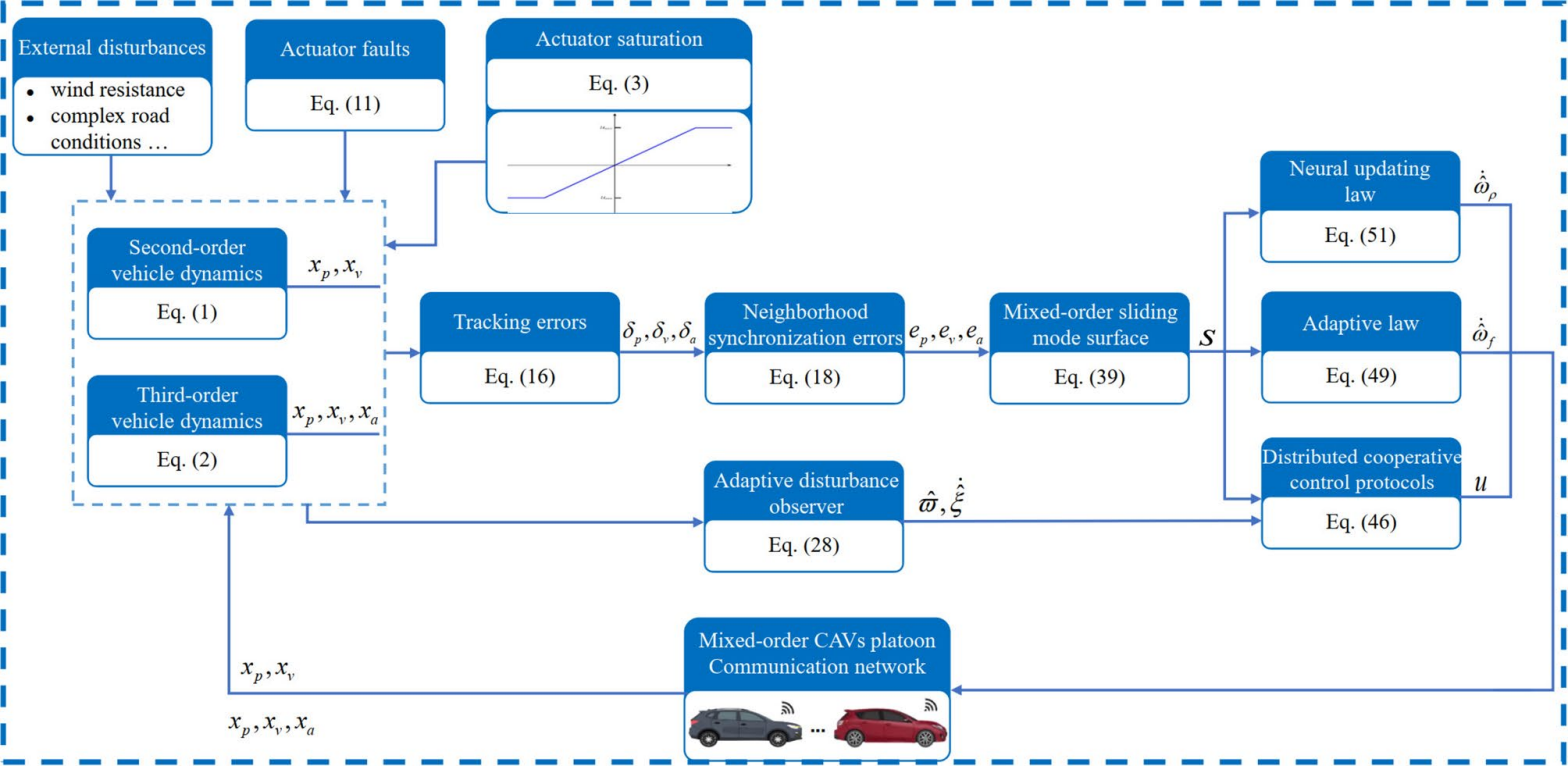
The design block diagram of the proposed adaptive fault-tolerant control method.

The novel adaptive fault-tolerant control method based on the developed ADOB and NNs adaptive mechanism is proposed to ensure stability and achieve the control goals in this section. To better illustrate the structure of the proposed adaptive fault-tolerant control method, the block diagram is provided in Fig. 2.

#### Assumption 3

(^1^) The actuator efficiency factor $\boldsymbol{\rho }$ is bounded by $\left\| \boldsymbol{\rho }\right\| \le \rho _{M}$ with constant $\rho _{M} > 0$. The $\boldsymbol{\vartheta }$ is bounded by $\left\| \boldsymbol{\vartheta }\right\| \le \vartheta _{M}$ with constant $\vartheta _{M} > 0$. The control input $\boldsymbol{u}$ is bounded by $\left\| \boldsymbol{u}\right\| \le u_{M}$ with constant $u_{M} > 0$. The leader’s state ${x}_{p,0}$, ${x}_{v,0}$ and ${x}_{a,0}$ are bounded; there exists $x_{p,0M}>0$, $x_{v,0M}>0$, and $x_{a,0M}>0$ such that $|x_{p,0}| \le x_{p,0M}$, $|x_{v,0}| \le x_{v,0M}$, and $|x_{a,0}| \le x_{a,0M}$. Unknown nonlinear function $f_0\left( {\bar{\boldsymbol{x}}_0,t}\right)$ is bounded by $|f_0\left( {\bar{\boldsymbol{x}}_0,t}\right) | \le f_{0M}$ with constant $f_{0M}>0$. The nonlinear unknown uncertainties $\boldsymbol{f}$ is bounded by $\left\| \boldsymbol{f}\right\| \le f_{M}$ with constant $f_{M} > 0$. The ADOB estimation error $\boldsymbol{\varepsilon }_{\varpi }$ is bounded by $\Vert \boldsymbol{\varepsilon }_{\varpi } \Vert \le \varepsilon _{\varpi M}$ with constant $\varepsilon _{\varpi M} > 0$.

#### Remark 4

The bounds involved in Assumption 2 and 3 will only be used in stability analysis, and will not be applied in control protocols design, which means that the values of these bounds do not need to be known.

Define the distributed mixed-order sliding mode surface as

39 $$\begin{aligned} s_i= {\left\{ \begin{array}{ll} \varphi _{1,i}e_{p,i}+e_{v,i},& i\in M_1 \\ \varphi _{2,i}e_{p,i}+\varphi _{3,i}e_{v,i}+e_{a,i},& i\in M_2 \end{array}\right. } \end{aligned}$$

where $\varphi _{1,i}>0$, $\varphi _{2,i}>0$, and $\varphi _{3,i}>0$ are the design sliding mode parameters and are selected such that the polynomial ${\gamma ^2}+{\varphi _{3,i}}{\gamma } + {\varphi _{2,i}}$ is Hurwitz for $\forall i\in M$.

Globally, (39) can be expressed as

40 $$\begin{aligned} \boldsymbol{s}&=\boldsymbol{s}_{m_{1}}+\boldsymbol{s}_{m_{2}} \nonumber \\ \boldsymbol{s}_{m_{1}}&=\boldsymbol{\Psi }_1\boldsymbol{e}_p^{M_1}+\boldsymbol{\boldsymbol{e}}_v^{M_1} \nonumber \\ \boldsymbol{s}_{m_{2}}&=\boldsymbol{\Psi }_{2}\boldsymbol{e}_{p}^{M_{2}}+\boldsymbol{\Psi }_{3}\boldsymbol{e}_{v}^{M_{2}}+\boldsymbol{e}_{a} \end{aligned}$$

where $$\boldsymbol{\Psi }_1=diag\left\{ {\textbf {0}}_{1\times m_2},\varphi _{1,1+m_2},\varphi _{1,2+m_2},\ldots ,\varphi _{1,m}\right\} \in \mathbb {R}^{m\times m}$$, $$\boldsymbol{\Psi }_2=diag\big \{ \varphi _{2,1},\varphi _{2,2},\ldots ,\varphi _{2,m_2}, {\textbf {0}}_{1\times (m-m_2)}\big \} \in \mathbb {R}^{m\times m}$$, $$\boldsymbol{\Psi }_3=diag\left\{ \varphi _{3,1},\varphi _{3,2},\ldots ,\varphi _{3,m_2},{\textbf {0}}_{1\times (m-m_2)}\right\} \in \mathbb {R}^{m\times m}$$.

Differentiating (40), we have

41 $$\begin{aligned} \dot{\boldsymbol{s}}&=\dot{\boldsymbol{s}}_{m_{1}}+\dot{\boldsymbol{s}}_{m_{2}} \nonumber \\ \dot{\boldsymbol{s}}_{m_{1}}&=\boldsymbol{\Psi }_1 \dot{\boldsymbol{e}}_p^{M_1}+\dot{\boldsymbol{e}}_v^{M_1} =\boldsymbol{\Psi }_1{\boldsymbol{e}}_v^{M_1}-(\boldsymbol{L}+\boldsymbol{B})(\dot{\boldsymbol{x}}^{M_1}_{v}-\dot{x}_{v,0} {\textbf {1}}^{M_1}) \nonumber \\&=\boldsymbol{\Psi }_1{\boldsymbol{e}}_v^{M_1}-(\boldsymbol{L}+\boldsymbol{B})({\boldsymbol{\vartheta }^{M_1}}\boldsymbol{\rho }^{M_1}{\boldsymbol{u}}^{M_1} + \boldsymbol{f}^{M_1} + {\boldsymbol{\varpi }}^{M_1}-{x}_{a,0} {\textbf {1}}^{M_1}) \nonumber \\ \dot{\boldsymbol{s}}_{m_{2}}&=\boldsymbol{\Psi }_{2} \dot{\boldsymbol{e}}_{p}^{M_{2}}+\boldsymbol{\Psi }_{3} \dot{\boldsymbol{e}}_{v}^{M_{2}}+\dot{\boldsymbol{e}}_{a}\nonumber \\&=\boldsymbol{\Psi }_{2} {\boldsymbol{e}}_{v}^{M_{2}}+\boldsymbol{\Psi }_{3} {\boldsymbol{e}}_{a}^{M_{2}}-(\boldsymbol{L}+\boldsymbol{B})({\boldsymbol{\vartheta }^{M_2}}{\boldsymbol{\rho }}^{M_2}{\boldsymbol{u}}^{M_2} + \boldsymbol{f}^{M_2} + {\boldsymbol{\varpi }}^{M_2}-f_0{\textbf {1}}^{M_2}) \end{aligned}$$

Define the auxiliary state matrices as

42 $$\begin{aligned}&\boldsymbol{E}_1=\begin{bmatrix}\boldsymbol{e}_p \ \boldsymbol{e}_v\end{bmatrix} \nonumber \\&\boldsymbol{E}_{2}=\dot{\boldsymbol{E}}_{1}=\begin{bmatrix}\dot{\boldsymbol{e}}_p \ \dot{\boldsymbol{e}}_v\end{bmatrix} \end{aligned}$$

where $$\boldsymbol{e}_{p}=\boldsymbol{e}_{p}^{M_{1}}+\boldsymbol{e}_{p}^{M_{2}}$$, $$\boldsymbol{e}_{v}=\boldsymbol{e}_{v}^{M_{1}}+\boldsymbol{e}_{v}^{M_{2}}$$.

Then, we have

43 $$\begin{aligned} \dot{\boldsymbol{E}}_{1}^{M_{1}}&=\begin{bmatrix}\dot{\boldsymbol{e}}_p^{M_1} \ \dot{\boldsymbol{e}}_v^{M_1}\end{bmatrix} =\begin{bmatrix}\boldsymbol{e}_p^{M_1} \ {\boldsymbol{e}}_v^{M_1}\end{bmatrix}\begin{bmatrix} {\textbf {0}}_{m}& \boldsymbol{I}_{m}\\ bm{\Psi }_1& -\boldsymbol{I}_{m}\end{bmatrix}^T + \left[ \boldsymbol{s}_{m_1}-(\boldsymbol{L}+\boldsymbol{B})(\dot{\boldsymbol{x}}_v^{M_1}-x_{a,0} {\textbf {1}}^{M_1})\right] \begin{bmatrix} {\textbf {0}}_{m} \\ \boldsymbol{I}_{m}\end{bmatrix}^T \nonumber \\ \dot{\boldsymbol{E}}_{1}^{M_{2}}&=\begin{bmatrix}\dot{\boldsymbol{e}}_p^{M_2} \ \dot{\boldsymbol{e}}_v^{M_2}\end{bmatrix} =\begin{bmatrix}\boldsymbol{e}_v^{M_2} \ {\boldsymbol{e}}_a\end{bmatrix} =\begin{bmatrix}{\boldsymbol{e}}_p^{M_2} \ {\boldsymbol{e}}_v^{M_2}\end{bmatrix} \begin{bmatrix} {\textbf {0}}_{m}& \boldsymbol{I}_{m}\\ bm{\Psi }_2& -\boldsymbol{\Psi }_3\end{bmatrix}^T + \boldsymbol{s}_{m_2}\begin{bmatrix} {\textbf {0}}_{m} \\ \boldsymbol{I}_{m}\end{bmatrix}^T \end{aligned}$$

According to (42) and (43), we have

44 $$\begin{aligned}&\boldsymbol{E}_{2}^{M_{1}}=\dot{\boldsymbol{E}}_{1}^{M_{1}}=\boldsymbol{E}_{1}^{M_{1}}\boldsymbol{\Lambda }_{1}^{T}+(\boldsymbol{s}_{m_{1}}-\boldsymbol{\boldsymbol{\Upsilon }})\boldsymbol{\Theta }^{T}_1 \nonumber \\&\boldsymbol{E}_{2}^{M_{2}}=\dot{\boldsymbol{E}}_{1}^{M_{2}}=\boldsymbol{E}_{1}^{M_{2}}\boldsymbol{\Lambda }_{2}^{T}+\boldsymbol{s}_{m_{2}}\boldsymbol{\Theta }^{T}_2 \end{aligned}$$

where $$\boldsymbol{\Lambda }_{1}=\begin{bmatrix} {\textbf {0}}_{m}& \boldsymbol{I}_{m}\\ bm{\Psi }_1& -\boldsymbol{I}_{m}\end{bmatrix} \in \mathbb {R}^{2m\times 2m}$$, $$\boldsymbol{\Lambda }_{2}=\begin{bmatrix} {\textbf {0}}_{m}& \boldsymbol{I}_{m}\\ bm{\Psi }_2& -\boldsymbol{\Psi }_3\end{bmatrix} \in \mathbb {R}^{2m\times 2m}$$, $$\boldsymbol{\Upsilon }=(\boldsymbol{L}+\boldsymbol{B})(\dot{\boldsymbol{x}}_v^{M_1}-x_{a,0} {\textbf {1}}^{M_1}) \in \mathbb {R}^{m}$$, $$\boldsymbol{\Theta }_1 =\begin{bmatrix} {\textbf {0}}_{m} \\ \boldsymbol{I}_{m}\end{bmatrix} \in \mathbb {R}^{2m\times m}$$, $$\boldsymbol{\Theta }_2 =\begin{bmatrix} {\textbf {0}}_{m} \\ \boldsymbol{I}_{m}\end{bmatrix} \in \mathbb {R}^{2m\times m}$$.

As $\boldsymbol{\Lambda }_{1}$ and $\boldsymbol{\Lambda }_{2}$ are Hurwitz, given any $\mu _1>0$ and $\mu _2>0$, there exist positive definite matrices $\boldsymbol{P}_1$ and $\boldsymbol{P}_2$ that satisfy

45 $$\begin{aligned} \boldsymbol{\Lambda }_{1}^T \boldsymbol{P}_1+\boldsymbol{P}_1\boldsymbol{\Lambda }_{1}=-\mu _1 \boldsymbol{I}_{2m} \nonumber \\ \boldsymbol{\Lambda }_{2}^T \boldsymbol{P}_2+\boldsymbol{P}_2\boldsymbol{\Lambda }_{2}=-\mu _2 \boldsymbol{I}_{2m} \end{aligned}$$

The novel distributed fault-tolerant cooperative control protocols for the mixed-order CAVs platoon are designed as

46 $$\begin{aligned} u_i(t)&=\frac{1}{{\vartheta } _i{\hat{\rho }}_i(t)}\left[ cs_i(t)-{\hat{\omega }}_{f,i}^T h_{f,i}(\bar{\boldsymbol{x}}_i) - \hat{\varpi }_i(t)\right] +\frac{\varphi _{1,i}e_{v,i}}{{\vartheta } _i(d_i+b_i){\hat{\rho }}_i(t)}, \ i\in M_1 \nonumber \\ u_i(t)&=\frac{1}{{\vartheta } _i{\hat{\rho }}_i(t)}\left[ cs_i(t)-{\hat{\omega }}_{f,i}^T h_{f,i}(\bar{\boldsymbol{x}}_i) - \hat{\varpi }_i(t)\right] +\frac{\varphi _{2,i}e_{v,i}+\varphi _{3,i}e_{a,i}}{{\vartheta } _i(d_i+b_i){\hat{\rho }}_i(t)}, \ i\in M_2 \end{aligned}$$

where *c* is the control gain.

Globally, based on (25), (46) becomes

47 $$\begin{aligned} \boldsymbol{\vartheta }^{M_1}\hat{\boldsymbol{\rho }}^{M_1}{\boldsymbol{u}}^{M_1}&=c\boldsymbol{s}^{M_1}-(\hat{\boldsymbol{\omega }}^{M_1}_f)^T \boldsymbol{h}^{M_1}_f - \hat{\boldsymbol{\varpi }}^{M_1} +(\boldsymbol{D}+\boldsymbol{B})^{-1} \boldsymbol{\Psi }_1 e_v^{M_1} \nonumber \\ \boldsymbol{\vartheta }^{M_2}\hat{\boldsymbol{\rho }}^{M_2}{\boldsymbol{u}}^{M_2}&=c\boldsymbol{s}^{M_2}-(\hat{\boldsymbol{\omega }}^{M_2}_f)^T \boldsymbol{h}^{M_2}_f - \hat{\boldsymbol{\varpi }}^{M_2} +(\boldsymbol{D}+\boldsymbol{B})^{-1} (\boldsymbol{\Psi }_2 \boldsymbol{e}_v^{M_2}+\boldsymbol{\Psi }_3 \boldsymbol{e}_a) \end{aligned}$$

Gain *c* satisfies

48 $$\begin{aligned} c>\frac{1}{\underline{\sigma }(\boldsymbol{Q})}\{\frac{2\nu ^2}{\varsigma _{f}}+\frac{2\theta ^2}{\varsigma _{\rho }} +\frac{4\left[ -\frac{1}{2}\overline{\sigma }(\boldsymbol{P}_M)+\iota \right] ^2}{\underline{\mu }}+2\chi \} \end{aligned}$$

where $\nu =-\frac{1}{2}h_{f M}{\overline{\sigma }}(\boldsymbol{P}){\overline{\sigma }}(\boldsymbol{A})$, $\theta =-\frac{1}{2}h_{\rho M}{u_M}{\overline{\sigma }}(\boldsymbol{P}){\overline{\sigma }}(\boldsymbol{A}){\boldsymbol{\vartheta }}$, $\overline{\sigma }(\boldsymbol{P}_M)=\max \{\overline{\sigma }(\boldsymbol{P}_1),\overline{\sigma }(\boldsymbol{P}_2)\}$, $\iota =-\frac{1}{2}\frac{\overline{\sigma }(\boldsymbol{P})\overline{\sigma }(\boldsymbol{A})}{\underline{\sigma }(\boldsymbol{D}+\boldsymbol{B})} \left[ \overline{\sigma }(\boldsymbol{\Psi }_{1}^2)+\overline{\sigma }(\boldsymbol{\Psi }_{2})\overline{\sigma }(\boldsymbol{\Psi }_{3})+\overline{\sigma }(\boldsymbol{\Psi }^2_{3})+\overline{\sigma }(\boldsymbol{\Psi }_{2})\right]$, $\chi =\frac{\overline{\sigma }(\boldsymbol{P})\overline{\sigma }(\boldsymbol{A})}{\underline{\sigma }(\boldsymbol{D}+\boldsymbol{B})} \left[ \overline{\sigma }(\boldsymbol{\Psi }_{1})+\overline{\sigma }(\boldsymbol{\Psi }_{3})\right]$, $\underline{\mu }=\min \{\mu _1,\mu _2\}$, $\varsigma _{f}$ and $\varsigma _{\rho }$ are defined in (49) and (51), respectively.

The adaptive law is designed as

49 $$\begin{aligned} \dot{\hat{\boldsymbol{\omega }}}_{f,i}&=-\Gamma _{f,i}\left[ \boldsymbol{h}_{f,i}(\bar{\boldsymbol{x}}_i)s^T_i(t) p_i(d_i+b_i) +\varsigma _{f}{\hat{\boldsymbol{\omega }}}_{f,i}\right] , \ i\in M \end{aligned}$$

where $\Gamma _{f,i} > 0$ is the parameter to be designed, $\varsigma _{f}>0$ is the positive gain.

Globally, we have

50 $$\begin{aligned} \dot{\hat{\boldsymbol{\omega }}}_{f}&=-\boldsymbol{\Gamma }_{f}\left[ \boldsymbol{h}_{f}\boldsymbol{s}^T \boldsymbol{P}(\boldsymbol{D}+\boldsymbol{B})+\varsigma _{f}{\hat{\boldsymbol{\omega }}}_{f}\right] \end{aligned}$$

where $$\boldsymbol{\Gamma }_f=diag\left\{ \Gamma _{f,1},\Gamma _{f,2},\ldots ,\Gamma _{f,m}\right\} \in \mathbb {R}^{m\cdot N_s\times m\cdot N_s}$$.

Design the neural updating law as

51 $$\begin{aligned} \dot{{\hat{\omega }}}_{\rho ,i}= {\left\{ \begin{array}{ll} -\Gamma _{\rho ,i}\left[ \boldsymbol{h}_{\rho ,i}(\bar{\boldsymbol{x}}_i)u_i(t) s^T_i(t) p_i(d_i+b_i){{\vartheta }_i} +\varsigma _{\rho }{\hat{\boldsymbol{\omega }}}_{\rho ,i}\right] & , \textrm{if} \ \hat{{\rho }}_i>\underline{\rho }_i \\ -\Gamma _{\rho ,i}\left[ \boldsymbol{h}_{\rho ,i}(\bar{\boldsymbol{x}}_i)u_i(t) s^T_i(t) p_i(d_i+b_i){{\vartheta }_i} +\varsigma _{\rho }{\hat{\boldsymbol{\omega }}}_{\rho ,i}\right] & , \textrm{if} \ \hat{{\rho }}_i=\underline{\rho }_i \\ & \hspace{-25mm} \textrm{and} \ \left[ \boldsymbol{h}_{\rho ,i}(\bar{\boldsymbol{x}}_i)u_i(t) s^T_i(t) p_i(d_i+b_i){{\vartheta }_i} +\varsigma _{\rho }{\hat{\boldsymbol{\omega }}}_{\rho ,i} \right] <0 \\ 0 & ,\textrm{if} \ \hat{{\rho }}_i=\underline{\rho }_i \\ & \hspace{-25mm} \textrm{and} \ \left[ \boldsymbol{h}_{\rho ,i}(\bar{\boldsymbol{x}}_i)u_i(t) s^T_i(t) p_i(d_i+b_i){{\vartheta }_i} +\varsigma _{\rho }{\hat{\boldsymbol{\omega }}}_{\rho ,i}\right] \ge 0 \end{array}\right. } \end{aligned}$$

where $\Gamma _{\rho ,i}>0$ is the parameter to be designed, $\varsigma _{\rho }>0$ is the positive gain.

Globally, we have

52 $$\begin{aligned} \dot{\hat{\boldsymbol{\omega }}}_{\rho }= {\left\{ \begin{array}{ll} -\boldsymbol{\Gamma }_{\rho }\left[ \boldsymbol{h}_{\rho }\boldsymbol{u} \boldsymbol{s}^T \boldsymbol{P}(\boldsymbol{D}+\boldsymbol{B}){\boldsymbol{\vartheta }} +\varsigma _{\rho }{\hat{\boldsymbol{\omega }}}_{\rho }\right] & ,\textrm{if} \ \hat{\boldsymbol{\rho }}>\underline{\boldsymbol{\rho }} \\ -\boldsymbol{\Gamma }_{\rho }\left[ \boldsymbol{h}_{\rho }\boldsymbol{u} \boldsymbol{s}^T \boldsymbol{P}(\boldsymbol{D}+\boldsymbol{B}){\boldsymbol{\vartheta }} +\varsigma _{\rho }{\hat{\boldsymbol{\omega }}}_{\rho }\right] & ,\textrm{if} \ \hat{\boldsymbol{\rho }}=\underline{\boldsymbol{\rho }} \ \textrm{and} \ \left[ \boldsymbol{h}_{\rho }\boldsymbol{u} \boldsymbol{s}^T \boldsymbol{P}(\boldsymbol{D}+\boldsymbol{B}){\boldsymbol{\vartheta }}+\varsigma _{\rho }{\hat{\boldsymbol{\omega }}}_{\rho }\right] <0 \\ 0 & , \textrm{if} \ \hat{\boldsymbol{\rho }}=\underline{\boldsymbol{\rho }} \ \textrm{and} \ \left[ \boldsymbol{h}_{\rho }\boldsymbol{u} \boldsymbol{s}^T \boldsymbol{P}(\boldsymbol{D}+\boldsymbol{B}){\boldsymbol{\vartheta }}+\varsigma _{\rho }{\hat{\boldsymbol{\omega }}}_{\rho }\right] \ge 0 \end{array}\right. } \end{aligned}$$

where $$\boldsymbol{\Gamma }_\rho =diag\left\{ \Gamma _{\rho ,1},\Gamma _{\rho ,2},\ldots ,\Gamma _{\rho ,m}\right\} \in \mathbb {R}^{m\cdot N_s\times m\cdot N_s}$$.

### Stability analysis

#### Theorem 2

For the leader (14) and mixed-order CAVs (13) under Assumption 2 and 3, the designed ADOB (28), distributed fault-tolerant cooperative control protocols (46), adaptive law (49), and neural updating law (51) can ensure that the tracking errors $\delta _{p,i}, i\in M$, $\delta _{v,i}, i\in M$, and $\delta _{a,i}, i\in M_2$ are CUUB, the synchronization errors $e_{p,i}, i\in M$, $e_{v,i}, i\in M$, and $e_{a,i}, i\in M_2$ are bounded, and the states $x_{p,i}, i\in M$, $x_{v,i}, i\in M$, and $x_{a,i}, i\in M_2$ for $\forall t\ge t_0$ are bounded.

#### Proof

According to (41), we have

53 $$\begin{aligned} \dot{\boldsymbol{s}}_{m_{1}}&=\boldsymbol{\Psi }_1{\boldsymbol{e}}_v^{M_1}-(\boldsymbol{L}+\boldsymbol{B})\left[ {\boldsymbol{\vartheta }^{M_1}}{\boldsymbol{\rho }}^{M_1}{\boldsymbol{u}}^{M_1} + \boldsymbol{f}^{M_1} + {\boldsymbol{\varpi }}^{M_1}+ {\boldsymbol{\vartheta }^{M_1}}(\hat{\boldsymbol{\rho }}^{M_1}{\boldsymbol{u}}^{M_1} -\hat{\boldsymbol{\rho }}^{M_1}{\boldsymbol{u}}^{M_1}) -{x}_{a,0} {\textbf {1}}^{M_1}\right] \nonumber \\&=\boldsymbol{\Psi }_1{\boldsymbol{e}}_v^{M_1}-(\boldsymbol{L}+\boldsymbol{B})\left[ {\boldsymbol{\vartheta }^{M_1}}\tilde{\boldsymbol{\rho }}^{M_1}{\boldsymbol{u}}^{M_1} + \boldsymbol{f}^{M_1} + {\boldsymbol{\varpi }}^{M_1}+ {\boldsymbol{\vartheta }^{M_1}}\hat{\boldsymbol{\rho }}^{M_1}{\boldsymbol{u}}^{M_1}-{x}_{a,0} {\textbf {1}}^{M_1}\right] \nonumber \\ \dot{\boldsymbol{s}}_{m_{2}}&=\boldsymbol{\Psi }_{2} {\boldsymbol{e}}_{v}^{M_{2}}+\boldsymbol{\Psi }_{3} {\boldsymbol{e}}_{a}-(\boldsymbol{L}+\boldsymbol{B})\left[ {\boldsymbol{\vartheta }^{M_2}}{\boldsymbol{\rho }}^{M_2}{\boldsymbol{u}}^{M_2} + \boldsymbol{f}^{M_2} + {\boldsymbol{\varpi }}^{M_2} + {\boldsymbol{\vartheta }^{M_2}}({\hat{\boldsymbol{\rho }}^{M_2}{\boldsymbol{u}}^{M_2}-\hat{\boldsymbol{\rho }}^{M_2}{\boldsymbol{u}}^{M_2}}) -f_0{\textbf {1}}^{M_2}\right] \nonumber \\&=\boldsymbol{\Psi }_{2} {\boldsymbol{e}}_{v}^{M_{2}}+\boldsymbol{\Psi }_{3} {\boldsymbol{e}}_{a}-(\boldsymbol{L}+\boldsymbol{B}) \left[ {\boldsymbol{\vartheta }^{M_2}}\tilde{\boldsymbol{\rho }}^{M_2}{\boldsymbol{u}}^{M_2} + \boldsymbol{f}^{M_2}+{\boldsymbol{\varpi }}^{M_2} + {\boldsymbol{\vartheta }^{M_2}}{\hat{\boldsymbol{\rho }}^{M_2}{\boldsymbol{u}}^{M_2}} -f_0{\textbf {1}}^{M_2}\right] \end{aligned}$$

According to (24), (25), and (47), (53) becomes

54 $$\begin{aligned} \dot{\boldsymbol{s}}_{m_{1}}&=\boldsymbol{\Psi }_1{\boldsymbol{e}}_v^{M_1}-(\boldsymbol{L}+\boldsymbol{B})\big [{\boldsymbol{\vartheta }^{M_1}}(\tilde{\boldsymbol{\omega }}_{\rho }^{M_1})^T \boldsymbol{h}_{\rho }^{M_1} {\boldsymbol{u}}^{M_1}+ {\boldsymbol{\varepsilon }^{M_1}_{\rho }} + (\boldsymbol{\omega }_{f}^{M_1})^T \boldsymbol{h}_{f}^{M_1} + {\boldsymbol{\varepsilon }^{M_1}_{f}} \nonumber \\&\quad + {\boldsymbol{\varpi }}^{M_1}+c\boldsymbol{s}^{M_1}-(\hat{\boldsymbol{\omega }}^{M_1}_f)^T \boldsymbol{h}^{M_1}_f- \hat{\boldsymbol{\varpi }}^{M_1} + (\boldsymbol{D}+\boldsymbol{B})^{-1} \boldsymbol{\Psi }_1 {\boldsymbol{e}}_v^{M_1} -{x}_{a,0} {\textbf {1}}^{M_1}\big ] \nonumber \\&=-(\boldsymbol{L}+\boldsymbol{B})\big [ {\boldsymbol{\vartheta }^{M_1}}(\tilde{\boldsymbol{\omega }}_{\rho }^{M_1})^T \boldsymbol{h}_{\rho }^{M_1} {\boldsymbol{u}}^{M_1} + {\boldsymbol{\varepsilon }^{M_1}_{\rho }}+c\boldsymbol{s}^{M_1} + (\tilde{\boldsymbol{\omega }}_{f}^{M_1})^T \boldsymbol{h}_{f}^{M_1} + {\boldsymbol{\varepsilon }^{M_1}_{f}} \nonumber \\&\quad + \tilde{\boldsymbol{\varpi }}^{M_1} + {\boldsymbol{\varepsilon }^{M_1}_{\varpi }} -{x}_{a,0} {\textbf {1}}^{M_1}\big ] + \boldsymbol{A}(\boldsymbol{D}+\boldsymbol{B})^{-1} \boldsymbol{\Psi }_1 {\boldsymbol{e}}_v^{M_1} \nonumber \\ \dot{\boldsymbol{s}}_{m_{2}}&=\boldsymbol{\Psi }_{2} {\boldsymbol{e}}_{v}^{M_{2}}+\boldsymbol{\Psi }_{3} {\boldsymbol{e}}_{a}-(\boldsymbol{L}+\boldsymbol{B})\big [{\boldsymbol{\vartheta }^{M_2}}(\tilde{\boldsymbol{\omega }}_{\rho }^{M_2})^T \boldsymbol{h}_{\rho }^{M_2} {\boldsymbol{u}}^{M_2} + {\boldsymbol{\varepsilon }^{M_2}_{\rho }} + (\boldsymbol{\omega }_{f}^{M_2})^T \boldsymbol{h}_{f}^{M_1}+ {\boldsymbol{\varepsilon }^{M_2}_{f}} \nonumber \\&\quad + {\boldsymbol{\varpi }}^{M_2} + c\boldsymbol{s}^{M_2}-(\hat{\boldsymbol{\omega }}^{M_2}_f)^T \boldsymbol{h}^{M_2}_f + (\boldsymbol{D}+\boldsymbol{B})^{-1} (\boldsymbol{\Psi }_2 \boldsymbol{e}_v^{M_2}+\boldsymbol{\Psi }_3 \boldsymbol{e}_a) - \hat{\boldsymbol{\varpi }}^{M_2}-f_0{\textbf {1}}^{M_2}\big ] \nonumber \\&=-(\boldsymbol{L}+\boldsymbol{B})\big [ {\boldsymbol{\vartheta }^{M_2}}(\tilde{\boldsymbol{\omega }}_{\rho }^{M_2})^T \boldsymbol{h}_{\rho }^{M_2} {\boldsymbol{u}}^{M_2} + {\boldsymbol{\varepsilon }^{M_2}_{\rho }} + c\boldsymbol{s}^{M_2} + (\tilde{\boldsymbol{\omega }}_{f}^{M_2})^T \boldsymbol{h}_{f}^{M_2} + {\boldsymbol{\varepsilon }^{M_2}_{f}} \nonumber \\&\quad + \tilde{\boldsymbol{\varpi }}^{M_2} + {\boldsymbol{\varepsilon }^{M_2}_{\varpi }}-f_0{\textbf {1}}^{M_2}\big ] + \boldsymbol{A}(\boldsymbol{D}+\boldsymbol{B})^{-1} (\boldsymbol{\Psi }_{2} {\boldsymbol{e}}_{v}^{M_{2}}+\boldsymbol{\Psi }_{3} {\boldsymbol{e}}_{a}) \end{aligned}$$

where $\varepsilon _{\varpi }$ represents the ADOB estimation error.

Define the Lyapunov function candidate as

55 $$\begin{aligned} V&=V_s+V_{\omega }+V_{E} \end{aligned}$$

where $$V_{E}=\frac{1}{2}tr\left\{ \boldsymbol{E}_{1}^{M_{1}}\boldsymbol{P}_{1}(\boldsymbol{E}_{1}^{M_{1}})^{T}+\boldsymbol{E}_{1}^{M_{2}}\boldsymbol{P}_{2}(\boldsymbol{E}_{1}^{M_{2}})^{T}\right\}$$, $$V_{\omega }=\frac{1}{2}tr\left\{ \tilde{\boldsymbol{\omega }}_{\rho }^{T}\boldsymbol{\Gamma }_{\rho }^{-1}\tilde{\boldsymbol{\omega }}_{\rho }\right\} +\frac{1}{2}tr\left\{ \tilde{\boldsymbol{\omega }}_{f}^{T}\boldsymbol{\Gamma }_{f}^{-1}\tilde{\boldsymbol{\omega }}_{f}\right\}$$, $$V_s=\frac{1}{2}s^{T}Ps$$.

The differential of $V_s$ can be expressed as

56 $$\begin{aligned} \dot{V}_s&=\boldsymbol{s}^{T}P \dot{\boldsymbol{s}} =(\boldsymbol{s}^{M_1})^T\boldsymbol{P}^{M_1}\dot{\boldsymbol{s}}^{M_1}+(\boldsymbol{s}^{M_2})^T\boldsymbol{P}^{M_2}\dot{\boldsymbol{s}}^{M_2} \nonumber \\&=-(\boldsymbol{s}^{M_1})^T\boldsymbol{P}^{M_1}(\boldsymbol{L}+\boldsymbol{B})\big [ {\boldsymbol{\vartheta }^{M_1}}(\tilde{\boldsymbol{\omega }}_{\rho }^{M_1})^T \boldsymbol{h}_{\rho }^{M_1} {\boldsymbol{u}}^{M_1} + {\boldsymbol{\varepsilon }^{M_1}_{\rho }} +c\boldsymbol{s}^{M_1} + (\tilde{\boldsymbol{\omega }}_{f}^{M_1})^T \boldsymbol{h}_{f}^{M_1} + {\boldsymbol{\varepsilon }^{M_1}_{f}} \nonumber \\&\quad + \tilde{\boldsymbol{\varpi }}^{M_1} + {\boldsymbol{\varepsilon }^{M_1}_{\varpi }}-{x}_{a,0} {\textbf {1}}^{M_1}\big ]-(\boldsymbol{s}^{M_2})^T\boldsymbol{P}^{M_2}(\boldsymbol{L}+\boldsymbol{B})\big [ {\boldsymbol{\vartheta }^{M_2}}(\tilde{\boldsymbol{\omega }}_{\rho }^{M_2})^T \boldsymbol{h}_{\rho }^{M_2} {\boldsymbol{u}}^{M_2} + {\boldsymbol{\varepsilon }^{M_2}_{\rho }} + c\boldsymbol{s}^{M_2} \nonumber \\&\quad + (\tilde{\boldsymbol{\omega }}_{f}^{M_2})^T \boldsymbol{h}_{f}^{M_2} + {\boldsymbol{\varepsilon }^{M_2}_{f}} + \tilde{\boldsymbol{\varpi }}^{M_2} + {\boldsymbol{\varepsilon }^{M_2}_{\varpi }}-f_0{\textbf {1}}^{M_2}\big ] + (\boldsymbol{s}^{M_1})^T\boldsymbol{P}^{M_1}\boldsymbol{A}(\boldsymbol{D}+\boldsymbol{B})^{-1} \boldsymbol{\Psi }_1 {\boldsymbol{e}}_v^{M_1} \nonumber \\&\quad + (\boldsymbol{s}^{M_2})^T\boldsymbol{P}^{M_2}\boldsymbol{A}(\boldsymbol{D}+\boldsymbol{B})^{-1} (\boldsymbol{\Psi }_{2} {\boldsymbol{e}}_{v}^{M_{2}}+\boldsymbol{\Psi }_{3} {\boldsymbol{e}}_{a}) \nonumber \\&=-c\boldsymbol{s}^T\boldsymbol{P}(\boldsymbol{L}+\boldsymbol{B})\boldsymbol{s}-\boldsymbol{s}^T \boldsymbol{P}(\boldsymbol{D}+\boldsymbol{B}){\boldsymbol{\vartheta }}\tilde{\boldsymbol{\omega }}_{\rho }^T \boldsymbol{h}_{\rho }\boldsymbol{u} +\boldsymbol{s}^T \boldsymbol{P} \boldsymbol{A} {\boldsymbol{\vartheta }}\tilde{\boldsymbol{\omega }}_{\rho }^T \boldsymbol{h}_{\rho }\boldsymbol{u} -\boldsymbol{s}^T \boldsymbol{P}(\boldsymbol{D}+\boldsymbol{B})\tilde{\boldsymbol{\omega }}_{f}^T \boldsymbol{h}_{f} +\boldsymbol{s}^T \boldsymbol{P}\boldsymbol{A}\tilde{\boldsymbol{\omega }}_{f}^T \boldsymbol{h}_{f} \nonumber \\&\quad -(\boldsymbol{s}^{M_1})^T\boldsymbol{P}^{M_1}(\boldsymbol{L}+\boldsymbol{B})\left[ {\boldsymbol{\varepsilon }^{M_1}_{\rho }} + {\boldsymbol{\varepsilon }^{M_1}_{f}}+\tilde{\boldsymbol{\varpi }}^{M_1} +{\boldsymbol{\varepsilon }^{M_1}_{\varpi }}-{x}_{a,0} {\textbf {1}}^{M_1}\right] + (\boldsymbol{s}^{M_1})^T\boldsymbol{P}^{M_1}\boldsymbol{A}(\boldsymbol{D}+\boldsymbol{B})^{-1} \boldsymbol{\Psi }_1 {\boldsymbol{e}}_v^{M_1}\nonumber \\&\quad -(\boldsymbol{s}^{M_2})^T\boldsymbol{P}^{M_2}(\boldsymbol{L}+\boldsymbol{B})\left[ {\boldsymbol{\varepsilon }^{M_2}_{\rho }} +{\boldsymbol{\varepsilon }^{M_2}_{f}} +\tilde{\boldsymbol{\varpi }}^{M_2}+{\boldsymbol{\varepsilon }^{M_2}_{\varpi }}-{f}_{0} {\textbf {1}}^{M_2}\right] \nonumber \\&\quad + (\boldsymbol{s}^{M_2})^T\boldsymbol{P}^{M_2}\boldsymbol{A}(\boldsymbol{D}+\boldsymbol{B})^{-1} (\boldsymbol{\Psi }_{2} {\boldsymbol{e}}_{v}^{M_{2}}+\boldsymbol{\Psi }_{3} {\boldsymbol{e}}_{a}) \end{aligned}$$

Since $\boldsymbol{x}^T\boldsymbol{y}={tr}\left\{ \boldsymbol{y}\boldsymbol{x}^T\right\}$, considering Lemma 1, (56) becomes

57 $$\begin{aligned} \dot{V}_s&=-\frac{1}{2}c\boldsymbol{s}^T\boldsymbol{Q}\boldsymbol{s}-tr\left\{ \tilde{\boldsymbol{\omega }}_{\rho }^T \boldsymbol{h}_{\rho }\boldsymbol{u} \boldsymbol{s}^T \boldsymbol{P}(\boldsymbol{D}+\boldsymbol{B}){\boldsymbol{\vartheta }}\right\} +tr\left\{ \tilde{\boldsymbol{\omega }}_{\rho }^T \boldsymbol{h}_{\rho }\boldsymbol{u} \boldsymbol{s}^T \boldsymbol{P}\boldsymbol{A}{\boldsymbol{\vartheta }}\right\} -tr\left\{ \tilde{\boldsymbol{\omega }}_{f}^T \boldsymbol{h}_{f} \boldsymbol{s}^T \boldsymbol{P}(\boldsymbol{D}+\boldsymbol{B})\right\} \nonumber \\&\quad + tr\left\{ \tilde{\boldsymbol{\omega }}_{f}^T \boldsymbol{h}_{f} \boldsymbol{s}^T \boldsymbol{P}\boldsymbol{A}\right\} - (\boldsymbol{s}^{M_1})^T\boldsymbol{P}^{M_1}(\boldsymbol{L}+\boldsymbol{B})\left[ {\boldsymbol{\varepsilon }^{M_1}_{\rho }} + {\boldsymbol{\varepsilon }^{M_1}_{f}}+\tilde{\boldsymbol{\varpi }}^{M_1} +{\boldsymbol{\varepsilon }^{M_1}_{\varpi }}-{x}_{a,0} {\textbf {1}}^{M_1}\right] \nonumber \\&\quad -(\boldsymbol{s}^{M_2})^T\boldsymbol{P}^{M_2}(\boldsymbol{L}+\boldsymbol{B})\left[ {\boldsymbol{\varepsilon }^{M_2}_{\rho }} +{\boldsymbol{\varepsilon }^{M_2}_{f}} +\tilde{\boldsymbol{\varpi }}^{M_2}+{\boldsymbol{\varepsilon }^{M_2}_{\varpi }} -{f}_{0} {\textbf {1}}^{M_2}\right] \nonumber \\&\quad + (\boldsymbol{s}^{M_1})^T\boldsymbol{P}^{M_1}\boldsymbol{A}(\boldsymbol{D}+\boldsymbol{B})^{-1} \boldsymbol{\Psi }_1 {\boldsymbol{e}}_v^{M_1} + (\boldsymbol{s}^{M_2})^T\boldsymbol{P}^{M_2}\boldsymbol{A}(\boldsymbol{D}+\boldsymbol{B})^{-1} (\boldsymbol{\Psi }_{2} {\boldsymbol{e}}_{v}^{M_{2}}+\boldsymbol{\Psi }_{3} {\boldsymbol{e}}_{a}) \end{aligned}$$

According to (55), we have

58 $$\begin{aligned} \dot{V}_{\omega }&=tr\left\{ \tilde{\boldsymbol{\omega }}_{\rho }^{T}\boldsymbol{\Gamma }_{\rho }^{-1}\dot{\tilde{\boldsymbol{\omega }}}_{\rho }\right\} +tr\left\{ \tilde{\boldsymbol{\omega }}_{f}^{T}\boldsymbol{\Gamma }_{f}^{-1} \dot{\tilde{\boldsymbol{\omega }}}_{f}\right\} =tr\left\{ \tilde{\boldsymbol{\omega }}_{\rho }^{T}\boldsymbol{\Gamma }_{\rho }^{-1}(\dot{{\boldsymbol{\omega }}}_{\rho }-\dot{\hat{\boldsymbol{\omega }}}_{\rho })\right\} +tr\left\{ \tilde{\boldsymbol{\omega }}_{f}^{T}\boldsymbol{\Gamma }_{f}^{-1} (\dot{\boldsymbol{\omega }}_{f}-\dot{\hat{\boldsymbol{\omega }}}_{f})\right\} \nonumber \\&=-tr\left\{ \tilde{\boldsymbol{\omega }}_{\rho }^{T}\boldsymbol{\Gamma }_{\rho }^{-1}\dot{\hat{\boldsymbol{\omega }}}_{\rho }\right\} -tr\left\{ \tilde{\boldsymbol{\omega }}_{f}^{T}\boldsymbol{\Gamma }_{f}^{-1} \dot{\hat{\boldsymbol{\omega }}}_{f}\right\} \end{aligned}$$

According to (44), we have

59 $$\begin{aligned} \dot{V}_E&=tr\left\{ \boldsymbol{E}_2^{M_1}\boldsymbol{P}_1(\boldsymbol{E}_1^{M_1})^T\right\} +tr\left\{ \boldsymbol{E}_2^{M_2}\boldsymbol{P}_2(\boldsymbol{E}_1^{M_2})^T\right\} \nonumber \\&=tr\left\{ [\boldsymbol{E}_{1}^{M_{1}}\boldsymbol{\Lambda }_{1}^{T}+(\boldsymbol{s}_{m_{1}}-\boldsymbol{\Upsilon })\boldsymbol{\Theta } ^{T}_1]\boldsymbol{P}_1(\boldsymbol{E}_1^{M_1})^T\right\} +tr\left\{ (\boldsymbol{E}_{1}^{M_{2}}\boldsymbol{\Lambda }_{2}^{T}+\boldsymbol{s}_{m_{2}}\boldsymbol{\Theta } ^{T}_2) \boldsymbol{P}_2(\boldsymbol{E}_1^{M_2})^T\right\} \nonumber \\&=tr\left\{ \boldsymbol{E}_{1}^{M_{1}}\boldsymbol{\Lambda }_{1}^{T}\boldsymbol{P}_1(\boldsymbol{E}_1^{M_1})^T +\boldsymbol{E}_{1}^{M_{2}}\boldsymbol{\Lambda }_{2}^{T}\boldsymbol{P}_2(\boldsymbol{E}_1^{M_2})^T\right\} +tr\left\{ \boldsymbol{s}_{m_{1}}\boldsymbol{\Theta }_1 ^{T}\boldsymbol{P}_1(\boldsymbol{E}_1^{M_1})^T + \boldsymbol{s}_{m_{2}}\boldsymbol{\Theta } ^{T}_1 \boldsymbol{P}_2(\boldsymbol{E}_1^{M_2})^T\right\} \nonumber \\&\quad - tr\left\{ \boldsymbol{\Upsilon }\boldsymbol{\Theta }_2 ^{T}\boldsymbol{P}_1(\boldsymbol{E}_1^{M_1})^T\right\} \end{aligned}$$

Then, combining (57), (58), and (59), $\dot{V}$ is described as

60 $$\begin{aligned} \dot{V}&=-\frac{1}{2}c\boldsymbol{s}^T\boldsymbol{Q}\boldsymbol{s}-tr\left\{ \tilde{\boldsymbol{\omega }}_{\rho }^T \left[ \boldsymbol{h}_{\rho }\boldsymbol{u} \boldsymbol{s}^T \boldsymbol{P}(\boldsymbol{D}+\boldsymbol{B}){\boldsymbol{\vartheta }} +\boldsymbol{\Gamma }_{\rho }^{-1}\dot{\hat{\boldsymbol{\omega }}}_{\rho }\right] \right\} -tr\left\{ \tilde{\boldsymbol{\omega }}_{f}^T \left[ \boldsymbol{h}_{f} \boldsymbol{s}^T \boldsymbol{P}(\boldsymbol{D}+\boldsymbol{B})+\boldsymbol{\Gamma }_{f}^{-1} \dot{\hat{\boldsymbol{\omega }}}_{f}\right] \right\} \nonumber \\&\quad - (\boldsymbol{s}^{M_1})^T\boldsymbol{P}^{M_1}(\boldsymbol{L}+\boldsymbol{B})\left[ {\boldsymbol{\varepsilon }^{M_1}_{\rho }} + {\boldsymbol{\varepsilon }^{M_1}_{f}}+\tilde{\boldsymbol{\varpi }}^{M_1} +{\boldsymbol{\varepsilon }^{M_1}_{\varpi }}-{x}_{a,0} {\textbf {1}}^{M_1}\right] +tr\left\{ \tilde{\boldsymbol{\omega }}_{\rho }^T \boldsymbol{h}_{\rho }\boldsymbol{u} \boldsymbol{s}^T \boldsymbol{P}\boldsymbol{A}{\boldsymbol{\vartheta }}\right\} \nonumber \\&\quad - (\boldsymbol{s}^{M_2})^T\boldsymbol{P}^{M_2}(\boldsymbol{L}+\boldsymbol{B})\left[ {\boldsymbol{\varepsilon }^{M_2}_{\rho }} +{\boldsymbol{\varepsilon }^{M_2}_{f}} +\tilde{\boldsymbol{\varpi }}^{M_2} +{\boldsymbol{\varepsilon }^{M_2}_{\varpi }} -{f}_{0} {\textbf {1}}^{M_2}\right] +tr\left\{ \tilde{\boldsymbol{\omega }}_{f}^T \boldsymbol{h}_{f} \boldsymbol{s}^T \boldsymbol{P}\boldsymbol{A}\right\} \nonumber \\&\quad + (\boldsymbol{s}^{M_1})^T\boldsymbol{P}^{M_1}\boldsymbol{A}(\boldsymbol{D}+\boldsymbol{B})^{-1} \boldsymbol{\Psi }_1 {\boldsymbol{e}}_v^{M_1} + (\boldsymbol{s}^{M_2})^T\boldsymbol{P}^{M_2}\boldsymbol{A}(\boldsymbol{D}+\boldsymbol{B})^{-1} (\boldsymbol{\Psi }_{2} {\boldsymbol{e}}_{v}^{M_{2}}+\boldsymbol{\Psi }_{3} {\boldsymbol{e}}_{a}) \nonumber \\&\quad + tr\left\{ \boldsymbol{E}_{1}^{M_{1}}\boldsymbol{\Lambda }_{1}^{T}\boldsymbol{P}_1(\boldsymbol{E}_1^{M_1})^T +\boldsymbol{E}_{1}^{M_{2}}\boldsymbol{\Lambda }_{2}^{T}\boldsymbol{P}_2(\boldsymbol{E}_1^{M_2})^T\right\} \nonumber \\&\quad +tr\left\{ \boldsymbol{s}_{m_{1}}\boldsymbol{\Theta } ^{T}_1\boldsymbol{P}_1(\boldsymbol{E}_1^{M_1})^T + \boldsymbol{s}_{m_{2}}\boldsymbol{\Theta }_2 ^{T} \boldsymbol{P}_2(\boldsymbol{E}_1^{M_2})^T\right\} -tr\left\{ \boldsymbol{\Upsilon }\boldsymbol{\Theta } ^{T}_1 \boldsymbol{P}_1(\boldsymbol{E}_1^{M_1})^T\right\} \end{aligned}$$

According to (50), (52), and Theorem 1, (60) becomes

61 $$\begin{aligned} \dot{V}&=-\frac{1}{2}c\boldsymbol{s}^T\boldsymbol{Q}\boldsymbol{s} +tr\left\{ \tilde{\boldsymbol{\omega }}_{\rho }^T \boldsymbol{h}_{\rho }\boldsymbol{u} \boldsymbol{s}^T \boldsymbol{P}\boldsymbol{A}{\boldsymbol{\vartheta }}\right\} +tr\left\{ \tilde{\boldsymbol{\omega }}_{f}^T \boldsymbol{h}_{f} \boldsymbol{s}^T \boldsymbol{P}\boldsymbol{A}\right\} +tr\left\{ \tilde{\boldsymbol{\omega }}_{f}^T\varsigma _{f}{\hat{\boldsymbol{\omega }}}_{f}\right\} +tr\left\{ \tilde{\boldsymbol{\omega }}_{\rho }^T\varsigma _{\rho }{\hat{\boldsymbol{\omega }}}_{\rho }\right\} \nonumber \\&\quad - (\boldsymbol{s}^{M_1})^T\boldsymbol{P}^{M_1}(\boldsymbol{L}+\boldsymbol{B})\left[ {\boldsymbol{\varepsilon }^{M_1}_{\rho }} + {\boldsymbol{\varepsilon }^{M_1}_{f}}+{\boldsymbol{\varepsilon }^{M_1}_{\varpi }} -{x}_{a,0} {\textbf {1}}^{M_1}\right] + (\boldsymbol{s}^{M_1})^T\boldsymbol{P}^{M_1}\boldsymbol{A}(\boldsymbol{D}+\boldsymbol{B})^{-1} \boldsymbol{\Psi }_1 {\boldsymbol{e}}_v^{M_1} \nonumber \\&\quad - (\boldsymbol{s}^{M_2})^T\boldsymbol{P}^{M_2}(\boldsymbol{L}+\boldsymbol{B})\left[ {\boldsymbol{\varepsilon }^{M_2}_{\rho }} +{\boldsymbol{\varepsilon }^{M_2}_{f}}+{\boldsymbol{\varepsilon }^{M_2}_{\varpi }} -{f}_{0} {\textbf {1}}^{M_2}\right] + (\boldsymbol{s}^{M_2})^T\boldsymbol{P}^{M_2}\boldsymbol{A}(\boldsymbol{D}+\boldsymbol{B})^{-1} (\boldsymbol{\Psi }_{2} {\boldsymbol{e}}_{v}^{M_{2}}+\boldsymbol{\Psi }_{3} {\boldsymbol{e}}_{a}) \nonumber \\&\quad +tr\left\{ \boldsymbol{E}_{1}^{M_{1}}\boldsymbol{\Lambda }_{1}^{T}\boldsymbol{P}_1(\boldsymbol{E}_1^{M_1})^T +\boldsymbol{E}_{1}^{M_{2}}\boldsymbol{\Lambda }_{2}^{T}\boldsymbol{P}_2(\boldsymbol{E}_1^{M_2})^T\right\} \nonumber \\&\quad +tr\left\{ \boldsymbol{s}_{m_{1}}\boldsymbol{\Theta } ^{T}_1\boldsymbol{P}_1(\boldsymbol{E}_1^{M_1})^T + \boldsymbol{s}_{m_{2}}\boldsymbol{\Theta } ^{T}_2 \boldsymbol{P}_2(\boldsymbol{E}_1^{M_2})^T\right\} -tr\left\{ \boldsymbol{\Upsilon }\boldsymbol{\Theta } ^{T}_1\boldsymbol{P}_1(\boldsymbol{E}_1^{M_1})^T\right\} \\&\quad - {\left\{ \begin{array}{ll} 0 & ,\textrm{if} \ \hat{\boldsymbol{\rho }}>\underline{\boldsymbol{\rho }} \nonumber \\ 0 & ,\textrm{if} \ \hat{\boldsymbol{\rho }}=\underline{\boldsymbol{\rho }} \ \textrm{and} \ \left[ \boldsymbol{h}_{\rho }\boldsymbol{u} \boldsymbol{s}^T \boldsymbol{P}(\boldsymbol{D}+\boldsymbol{B}){\boldsymbol{\vartheta }}+\varsigma _{\rho }{\hat{\boldsymbol{\omega }}}_{\rho }\right] <0 \nonumber \\ tr\left\{ \tilde{\boldsymbol{\omega }}_{\rho }^T\left[ \boldsymbol{h}_{\rho }\boldsymbol{u} \boldsymbol{s}^T \boldsymbol{P}(\boldsymbol{D}+\boldsymbol{B}){\boldsymbol{\vartheta }}+\varsigma _{\rho }{\hat{\boldsymbol{\omega }}}_{\rho }\right] \right\} & ,\textrm{if} \ \hat{\boldsymbol{\rho }}=\underline{\boldsymbol{\rho }} \ \textrm{and} \ \left[ \boldsymbol{h}_{\rho }\boldsymbol{u} \boldsymbol{s}^T \boldsymbol{P}(\boldsymbol{D}+\boldsymbol{B}){\boldsymbol{\vartheta }}+\varsigma _{\rho }{\hat{\boldsymbol{\omega }}}_{\rho }\right] \ge 0 \end{array}\right. } \end{aligned}$$

Then, we have

62 $$\begin{aligned} \dot{V}&\le -\frac{1}{2}c\boldsymbol{s}^T\boldsymbol{Q}\boldsymbol{s} +\varsigma _{f}tr\left\{ \tilde{\boldsymbol{\omega }}_{f}^T ({{\boldsymbol{\omega }}}_{f}-{\tilde{\boldsymbol{\omega }}}_{f})\right\} +\varsigma _{\rho }tr\left\{ \tilde{\boldsymbol{\omega }}_{\rho }^T ({{\boldsymbol{\omega }}}_{\rho }-{\tilde{\boldsymbol{\omega }}}_{\rho })\right\} +tr\left\{ \tilde{\boldsymbol{\omega }}_{\rho }^T \boldsymbol{h}_{\rho }\boldsymbol{u} \boldsymbol{s}^T \boldsymbol{P}\boldsymbol{A}{\boldsymbol{\vartheta }}\right\} \nonumber \\&\quad +tr\left\{ \tilde{\boldsymbol{\omega }}_{f}^T \boldsymbol{h}_{f} \boldsymbol{s}^T \boldsymbol{P}\boldsymbol{A}\right\} -(\boldsymbol{s}^{M_1})^T\boldsymbol{P}^{M_1}(\boldsymbol{L}+\boldsymbol{B})\left[ {\boldsymbol{\varepsilon }^{M_1}_{\rho }} + {\boldsymbol{\varepsilon }^{M_1}_{f}}+{\boldsymbol{\varepsilon }^{M_1}_{\varpi }} -{x}_{a,0} {\textbf {1}}^{M_1}\right] \nonumber \\&\quad + (\boldsymbol{s}^{M_1})^T\boldsymbol{P}^{M_1}\boldsymbol{A}(\boldsymbol{D}+\boldsymbol{B})^{-1} \boldsymbol{\Psi }_1 \boldsymbol{s}_{m_1} -(\boldsymbol{s}^{M_2})^T\boldsymbol{P}^{M_2}(\boldsymbol{L}+\boldsymbol{B})\left[ {\boldsymbol{\varepsilon }^{M_2}_{\rho }} +{\boldsymbol{\varepsilon }^{M_2}_{f}}+{\boldsymbol{\varepsilon }^{M_2}_{\varpi }} -{f}_{0} {\textbf {1}}^{M_2}\right] \nonumber \\&\quad + (\boldsymbol{s}^{M_2})^T\boldsymbol{P}^{M_2}\boldsymbol{A}(\boldsymbol{D}+\boldsymbol{B})^{-1} \boldsymbol{\Psi }_3 \boldsymbol{s}_{m_2} -(\boldsymbol{s}^{M_1})^T\boldsymbol{P}^{M_1}\boldsymbol{A}(\boldsymbol{D}+\boldsymbol{B})^{-1} \boldsymbol{\Psi }^2_1 \boldsymbol{E}^{M_1}_1 \begin{bmatrix}1\\ 0\end{bmatrix} \nonumber \\&\quad -(\boldsymbol{s}^{M_2})^T\boldsymbol{P}^{M_2}\boldsymbol{A}(\boldsymbol{D}+\boldsymbol{B})^{-1} \boldsymbol{\Psi }_2\boldsymbol{\Psi }_3 \boldsymbol{E}^{M_2}_1 \begin{bmatrix}1\\ 0\end{bmatrix}-(\boldsymbol{s}^{M_2})^T\boldsymbol{P}^{M_2}\boldsymbol{A}(\boldsymbol{D}+\boldsymbol{B})^{-1} {\boldsymbol{\Psi }_3}^2 \boldsymbol{E}^{M_2}_1 \begin{bmatrix}0\\ 1\end{bmatrix} \nonumber \\&\quad + (\boldsymbol{s}^{M_2})^T\boldsymbol{P}^{M_2}\boldsymbol{A}(\boldsymbol{D}+\boldsymbol{B})^{-1} \boldsymbol{\Psi }_2 \boldsymbol{E}^{M_2}_1 \begin{bmatrix}0\\ 1\end{bmatrix} +tr\left\{ \boldsymbol{E}_{1}^{M_{1}}\boldsymbol{\Lambda }_{1}^{T}\boldsymbol{P}_1(\boldsymbol{E}_1^{M_1})^T +\boldsymbol{E}_{1}^{M_{2}}\boldsymbol{\Lambda }_{2}^{T}\boldsymbol{P}_2(\boldsymbol{E}_1^{M_2})^T\right\} \nonumber \\&\quad + tr\left\{ \boldsymbol{s}_{m_{1}}\boldsymbol{\Theta }_1^{T}\boldsymbol{P}_1(\boldsymbol{E}_1^{M_1})^T + \boldsymbol{s}_{m_{2}}\boldsymbol{\Theta }_2^{T} \boldsymbol{P}_2(\boldsymbol{E}_1^{M_2})^T\right\} - tr\left\{ \boldsymbol{\Upsilon }\boldsymbol{\Theta }_1^{T}\boldsymbol{P}_1(\boldsymbol{E}_1^{M_1})^T\right\} \end{aligned}$$

Based on Remark 2 and Assumption 2 and 3, taking norm on (62), we have

63 $$\begin{aligned} \dot{V}&\le -\frac{1}{2}c\underline{\sigma }(\boldsymbol{Q})\Vert \boldsymbol{s}\Vert ^2 +\varsigma _{f}{\omega }_{f M}\left\| \tilde{\boldsymbol{\omega }}_f\right\| _F - \varsigma _{f}\left\| \tilde{\boldsymbol{\omega }}_f\right\| ^2_F +\varsigma _{\rho }{\omega }_{\rho M}\left\| \tilde{\boldsymbol{\omega }}_\rho \right\| _F - \varsigma _{\rho }\left\| \tilde{\boldsymbol{\omega }}_\rho \right\| ^2_F \nonumber \\&\quad + h_{\rho M}{u_M}{\overline{\sigma }}(\boldsymbol{P}){\overline{\sigma }}(\boldsymbol{A}){\boldsymbol{\vartheta }} \Vert \boldsymbol{s}\Vert \left\| \tilde{\boldsymbol{\omega }}_\rho \right\| _F + h_{f M}{\overline{\sigma }}(\boldsymbol{P}){\overline{\sigma }}(\boldsymbol{A}) \Vert \boldsymbol{s}\Vert \left\| \tilde{\boldsymbol{\omega }}_f\right\| _F \nonumber \\&\quad +\overline{\sigma }(\boldsymbol{P})\overline{\sigma }(\boldsymbol{L}+\boldsymbol{B})\mathcal {F}_{M}\Vert \boldsymbol{s}\Vert +\frac{\overline{\sigma }(\boldsymbol{P})\overline{\sigma }(\boldsymbol{A})}{\underline{\sigma }(\boldsymbol{D}+\boldsymbol{B})} \left[ \overline{\sigma }(\boldsymbol{\Psi }_{1})+\overline{\sigma }(\boldsymbol{\Psi }_{3})\right] \Vert \boldsymbol{s}\Vert ^2 \nonumber \\&\quad +\frac{\overline{\sigma }(\boldsymbol{P})\overline{\sigma }(\boldsymbol{A})}{\underline{\sigma }(\boldsymbol{D}+\boldsymbol{B})} \left[ \overline{\sigma }(\boldsymbol{\Psi }_{1}^2)+\overline{\sigma }(\boldsymbol{\Psi }_{2})\overline{\sigma }(\boldsymbol{\Psi }_{3})+\overline{\sigma }(\boldsymbol{\Psi }^2_{3})+\overline{\sigma }(\boldsymbol{\Psi }_{2})\right] \Vert \boldsymbol{s}\Vert \Vert \boldsymbol{E}_1\Vert _F \nonumber \\&\quad - \frac{1}{2}\underline{\mu } \Vert \boldsymbol{E}_1\Vert _F^2+\overline{\sigma }(\boldsymbol{P}_M) \Vert \boldsymbol{s}\Vert \Vert \boldsymbol{E}_1\Vert _F +\overline{\sigma }(\boldsymbol{L}+\boldsymbol{B})\overline{\sigma }(\boldsymbol{P}_{M})\beta _{M} \Vert \boldsymbol{E}_{1}\Vert _{F} \end{aligned}$$

where $$\mathcal {F}_{M}=\varepsilon _{\rho M} +{\varepsilon _{fM}}+{\varepsilon _{\varpi M}} +{x}_{a,0M}+f_{0M}$$, $$\beta _{M}={\vartheta }_M\rho _M u_M + f_M + \xi +{x}_{a,0M}$$.

According to (48), (63) is described as

64 $$\begin{aligned} \dot{V}\le -\boldsymbol{\ell }^T {\boldsymbol{\kappa }} \boldsymbol{\ell }+{\boldsymbol{\zeta }}^T \boldsymbol{\ell }=-V_{\boldsymbol{\ell }}(\boldsymbol{\ell }) \end{aligned}$$

where $$\boldsymbol{\ell }= \left[ \Vert \boldsymbol{s}\Vert , \Vert \tilde{\boldsymbol{\omega }}_{f}\Vert _{F}, \Vert \tilde{\boldsymbol{\omega }}_{\rho }\Vert _{F}, \Vert \boldsymbol{E}_{1}\Vert _{F} \right] ^T \in \mathbb {R}^{4},{\boldsymbol{\zeta }} = \left[ \overline{\sigma }(\boldsymbol{P})\overline{\sigma }(\boldsymbol{L}+\boldsymbol{B})\mathcal {F}_M, \varsigma _{f}{\omega }_{f M}, \varsigma _{\rho }{\omega }_{\rho M}, \overline{\sigma }(\boldsymbol{L}+\boldsymbol{B})\overline{\sigma }(\boldsymbol{P}_{M})\beta _M \right] ^T$$

$$\in \mathbb {R}^{4}, {\boldsymbol{\kappa }} = \begin{bmatrix} \frac{1}{2}c\underline{\sigma }(\boldsymbol{Q})-\chi & \nu & \theta & -\frac{1}{2}\overline{\sigma }(\boldsymbol{P}_{M})+\iota \\ \nu & \varsigma _{f} & 0 & 0\\ \theta & 0 & \varsigma _{\rho } & 0\\ -\frac{1}{2}\overline{\sigma }(\boldsymbol{P}_{M})+\iota & 0 & 0 & \frac{\underline{\mu }}{2} \end{bmatrix} \in \mathbb {R}^{4 \times 4}$$.

Then, $V_{\boldsymbol{\ell }}(\boldsymbol{\ell })>0$ if the following conditions hold.

(1) $${\boldsymbol{\kappa }}>0$$.

(2) $$\Vert \boldsymbol{\ell }\Vert \ge \frac{\Vert {\boldsymbol{\zeta }}\Vert }{\underline{\sigma }({\boldsymbol{\kappa }})}$$

With Sylvester’s criterion, ${\boldsymbol{\kappa }}>0$ if

65 $$\begin{gathered} \frac{1}{2}c\underset{\raise0.3em\hbox{$\smash{\scriptscriptstyle-}$}}{\sigma } (\user2{Q}) - \chi > 0 \hfill \\ \left[ {\frac{1}{2}c\underset{\raise0.3em\hbox{$\smash{\scriptscriptstyle-}$}}{\sigma } (\user2{Q}) - \chi } \right]\varsigma _{f} - \nu ^{2} > 0 \hfill \\ \left[ {\frac{1}{2}c\underset{\raise0.3em\hbox{$\smash{\scriptscriptstyle-}$}}{\sigma } (\user2{Q}) - \chi } \right]\varsigma _{f} \varsigma _{\rho } - \nu ^{2} \varsigma _{\rho } - \theta ^{2} \varsigma _{f} > 0 \hfill \\ \frac{1}{2}\left[ {\frac{1}{2}c\underset{\raise0.3em\hbox{$\smash{\scriptscriptstyle-}$}}{\sigma } (\user2{Q}) - \chi } \right]\varsigma _{f} \varsigma _{\rho } \underset{\raise0.3em\hbox{$\smash{\scriptscriptstyle-}$}}{\mu } - \frac{1}{2}\nu ^{2} \varsigma _{\rho } \underset{\raise0.3em\hbox{$\smash{\scriptscriptstyle-}$}}{\mu } \hfill \\ - \frac{1}{2}\theta ^{2} \varsigma _{f} \underset{\raise0.3em\hbox{$\smash{\scriptscriptstyle-}$}}{\mu } - \left[ { - \frac{1}{2}\bar{\sigma }(\user2{P}_{M} ) + \iota } \right]^{2} \varsigma _{f} \varsigma _{\rho } > 0 \hfill \\ \end{gathered}$$

Then, we can obtain condition (48) from (65).

Define $$Y_n$$ as

66 $$\begin{aligned} Y_n=\frac{\Vert {\boldsymbol{\zeta }} \Vert }{\underline{\sigma }({\boldsymbol{\kappa }})}=\frac{\overline{\sigma }(\boldsymbol{P})\overline{\sigma }(\boldsymbol{L}+\boldsymbol{B})\mathcal {F}_M +\varsigma _{f}{\omega }_{f M} +\varsigma _{\rho }{\omega }_{\rho M} +\overline{\sigma }(\boldsymbol{L}+\boldsymbol{B})\overline{\sigma }(\boldsymbol{P}_{M})\beta _M}{\underline{\sigma }({\boldsymbol{\kappa }})} \end{aligned}$$

Then, $\Vert \boldsymbol{\ell } \Vert >\frac{\Vert {\boldsymbol{\zeta }} \Vert }{\underline{\sigma }({\boldsymbol{\kappa }})}$ holds if $\Vert \boldsymbol{\ell } \Vert \ge Y_n$. Thus, by condition (48), we have $\dot{V}\le -V_{\boldsymbol{\ell }}(\boldsymbol{\ell })$ with $V_{\boldsymbol{\ell }}(\boldsymbol{\ell })>0$.

Based on (55), one has

67 $$\begin{aligned} {\underline{\sigma }(\boldsymbol{R})}\Vert \boldsymbol{\ell }\Vert ^2 \le V \le {\overline{\sigma }(\boldsymbol{H})}\Vert \boldsymbol{\ell }\Vert ^2 \end{aligned}$$

where $$\boldsymbol{R}=diag\left\{ \frac{\underline{\sigma }(\boldsymbol{P})}{2},\frac{1}{2\overline{\sigma }(\boldsymbol{\Gamma }_{\rho })}, \frac{1}{2\overline{\sigma }(\boldsymbol{\Gamma }_{f})}, \frac{\underline{\sigma }(\boldsymbol{P}_M)}{2} \right\} \in \mathbb {R}^{4\times 4}$$, $$\boldsymbol{H}=diag\left\{ \frac{\overline{\sigma }(\boldsymbol{P})}{2},\frac{1}{2\underline{\sigma }(\boldsymbol{\Gamma }_{\rho })}, \frac{1}{2\underline{\sigma }(\boldsymbol{\Gamma }_{f})}, \frac{\overline{\sigma }(\boldsymbol{P}_M)}{2} \right\} \in \mathbb {R}^{4\times 4}$$.

According to Theorem 4.18 in^42^, for any $\boldsymbol{\ell }(t_0)$, we can find a $T_0$ that satisfies

68 $$\begin{aligned} \Vert \boldsymbol{\ell }(t)\Vert \le \sqrt{\frac{\overline{\sigma }(\boldsymbol{H})}{\underline{\sigma }(\boldsymbol{R})}} Y_n, \ \forall t\ge t_0+T_0. \end{aligned}$$

Define $U_{\boldsymbol{\ell }}=\min \limits _{\Vert {\boldsymbol{\ell }} \Vert \ge Y_n} V_{{\boldsymbol{\ell }}}({\boldsymbol{\ell }})$. Then, we have

$$\begin{aligned} T_0=\frac{V({t_0})-\overline{\sigma }(\boldsymbol{H}) Y^2_n}{U_{{\boldsymbol{\ell }}}} \end{aligned}$$

(68) indicates that $\boldsymbol{s}$ is finite-time bounded. Then, $s_i$ is ultimately bounded. With Lemma 3.3 in^37^ $\boldsymbol{e}_p$, $\boldsymbol{e}_v$, and $\boldsymbol{e}_a$ are finite-time bounded. By Lemma 2, $\boldsymbol{\delta }_p$, $\boldsymbol{\delta }_v$, and $\boldsymbol{\delta }_a$ are CUUB.

Then, the boundedness of $x_{p,i}(t)$, $x_{v,i}(t)$, and $x_{a,i}(t)$ for $\forall t\ge t_0$ can be proved as follows. From (64), one can obtain

69 $$\begin{aligned} \dot{V}\le -\underline{\sigma }({\boldsymbol{\kappa }})\Vert {\boldsymbol{\ell }}\Vert ^{2}+\Vert {\boldsymbol{\zeta }}\Vert \Vert {\boldsymbol{\ell }}\Vert \end{aligned}$$

By (67) and (69), we have

$$\begin{aligned} \frac{d}{dt}(\sqrt{V})\le -\frac{\underline{\sigma }({\boldsymbol{\kappa }})}{2\overline{\sigma }(\boldsymbol{H})}\sqrt{V} +\frac{\Vert {\boldsymbol{\zeta }}\Vert }{2\sqrt{\underline{\sigma }(\boldsymbol{R})}} \end{aligned}$$

Then, under Corollary 1.1 in^41^, *V*(*t*) is bounded for $\forall t\ge t_0$. From (55), we have $\Vert \boldsymbol{s}\Vert ^2\le \frac{2V(t)}{\underline{\sigma }(\boldsymbol{P})}$, which implies that $\boldsymbol{s}(t)$ for $\forall t\ge t_0$ is bounded. By Lemma 3.3 in^37^ and Lemma 2, we can get that $\boldsymbol{\delta }_p$, $\boldsymbol{\delta }_v$, and $\boldsymbol{\delta }_a$ are bounded. According to (16), (17), and Assumption 3, we can obtain that $x_{p,i}$, $x_{v,i}$ for $i \in M$ and $x_{a,i}$ for $i \in M_2$ are bounded. The proof of Theorem 2 is done. $\square$

## Numerical example

**Fig. 3:**
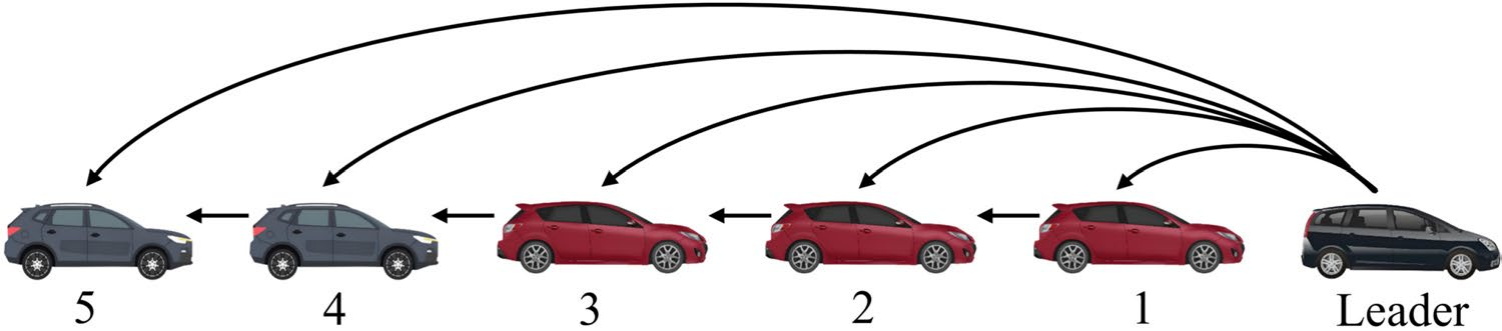
Communication topology of mixed-order CAVs platoon.

This section provides a numerical example to demonstrate the effectiveness and advantage of the developed control method. Consider the mixed-order CAVs platoon consisting of one leader and five following vehicles $(i=1,2,3,4,5)$, in which the following vehicles 1-3 have third-order dynamics and vehicles 4-5 have second-order dynamics as shown in Fig. 3. The leader’s initial states are $x_{p,0}(0) = 30m$, and $x_{v,0}(0) = 0.1m/s$, respectively. The leader’s acceleration trajectory is^17^

$$\begin{aligned} x_{a,0}(t)={\left\{ \begin{array}{ll} 0.5t & m/{s^2}, 0s \le t< 4s\\ 2 & m/{s^2}, 4s \le t< 8s\\ -0.5t+6 & m/{s^2}, 8s \le t < 12s\\ 0 & m/{s^2}, otherwise \end{array}\right. } \end{aligned}$$

The parameters of mixed-order CAVs platoon and controller are $\tau _i=0.5$, $\mathcal {R}_i=1.2kg/m^3$, $\mathcal {A}_i=2m^2$, $\mathcal {C}_i=0.35$, $\mathcal {D}_{ri}=5N$, $\mathcal {M}_i=1500kg$, $\epsilon _i=0.8sin(0.5t)+0.8cos(0.3t)$, $\rho _i(t)=0.75+0.25sin(0.1i \cdot t)$, $\underline{\rho }_i=0.001$, $r_i=0.01sin(t)$, ${\vartheta }_i=0.5$, $d_i=2.5$, ${\aleph }=1000$, $\alpha _{s}=50$, $\alpha _{\xi }=0.001$, $\varphi _{1,i}=200$, $\varphi _{2,i}=5$, $\varphi _{3,i}=20$, $c=300$, $\Gamma _{f,i}=5000$, $\varsigma _{f}=10^{-5}$, $\Gamma _{\rho ,i}=10^{-6}$, $\varsigma _{\rho }=10^{-4}$, $\boldsymbol{x}_{p}(0)=[27.5,25,22.5,20,17.5]$, $\boldsymbol{x}_{v}(0)=[0,0,0,0.1,0.1]$, and $\boldsymbol{x}_{a}(0)=[0,0,0,0,0]$ for $i=1,2,\ldots ,5$.

**Fig. 4:**
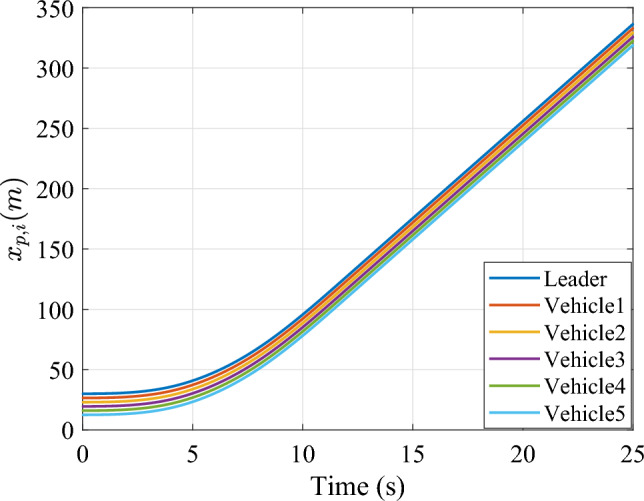
Position $$x_{p,i}(m)$$.

**Fig. 5:**
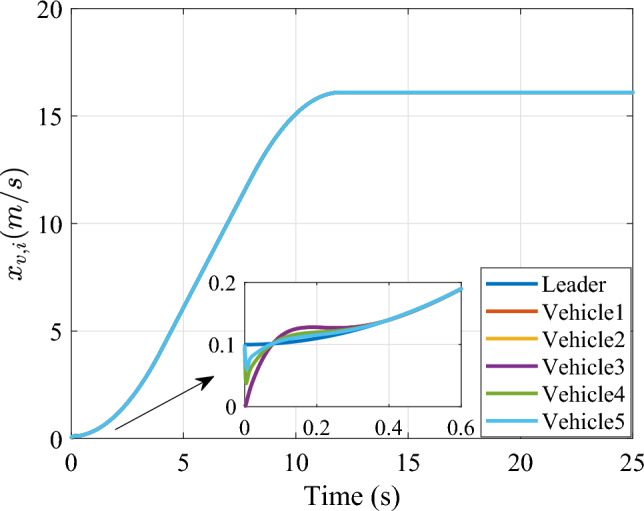
Velocity $$x_{v,i}(m/s)$$.

**Fig. 6:**
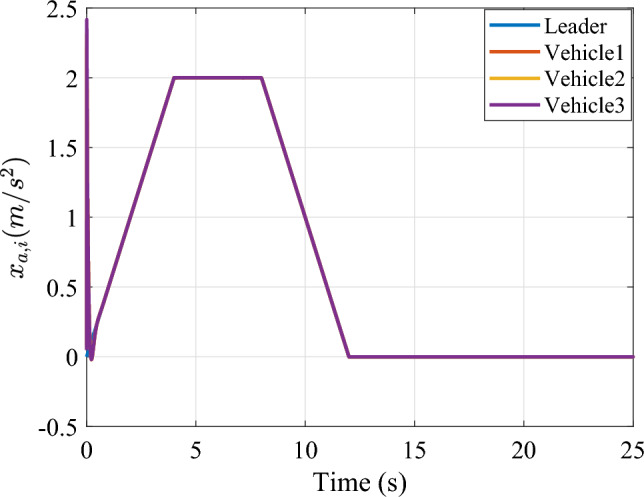
Acceleration $$x_{a,i}(m/s^2)$$.

**Fig. 7:**
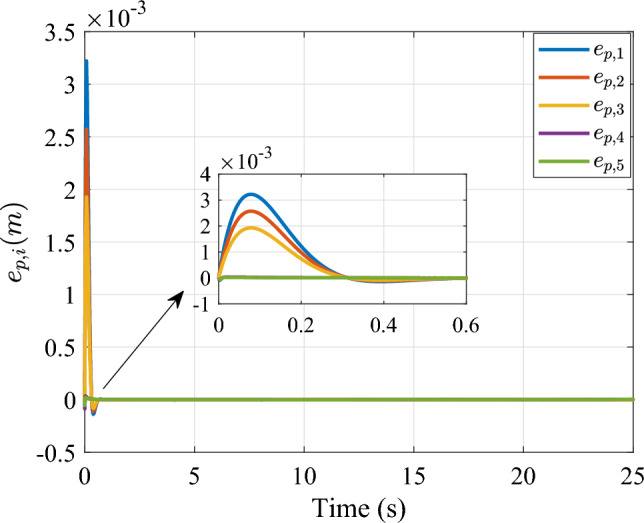
Spacing errors $$e_{p,i}(m)$$.

**Fig. 8:**
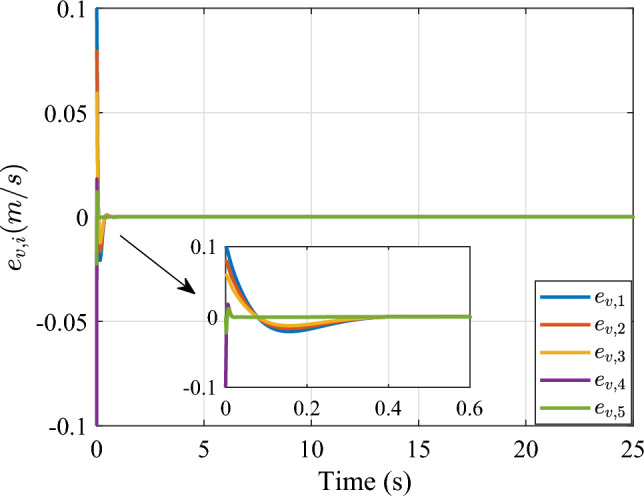
Velocity errors $$e_{v,i}(m/s)$$.

**Fig. 9:**
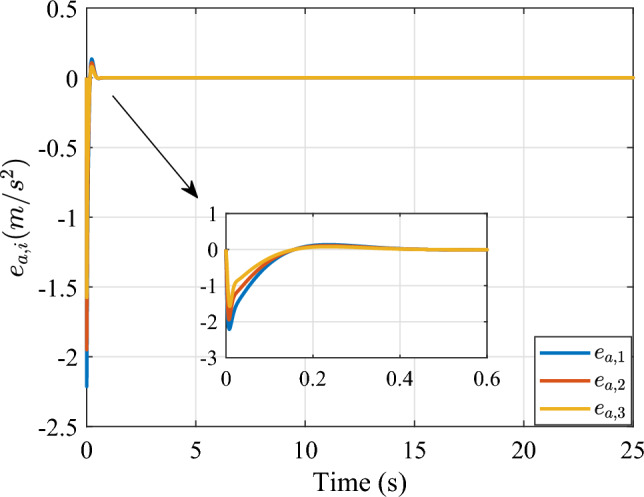
Acceleration errors $$e_{a,i}(m/s^2)$$.

**Fig. 10:**
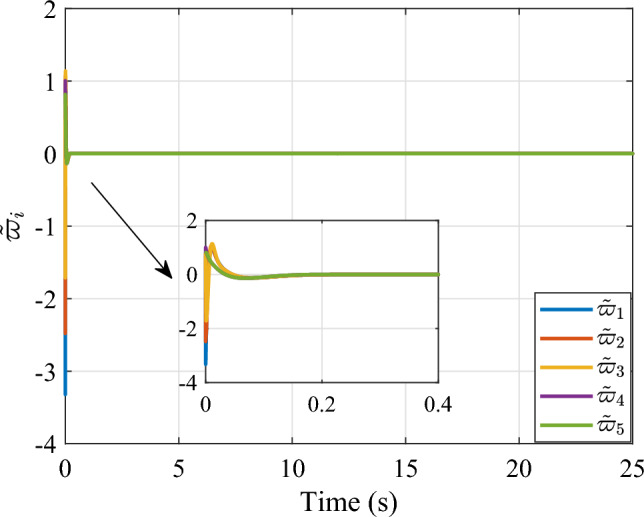
Estimated disturbance errors $\tilde{\varpi }_i$.

For the leader (14) and mixed-order CAVs (13) under the communication topology shown in Fig. 3, the designed ADOB (28), distributed fault-tolerant cooperative control protocols (46), adaptive law (49), and neural updating law (51) are implemented in the numerical example. The results of the numerical example are shown in Figs. 4, 5, 6, 7, 8, 9 and 10. The position curves of the vehicles are shown in Fig. 4. As there is no occurrence of intersecting or overlapping, it is indicated that the designed control method prevents collisions between adjacent vehicles and ensures safe operation of the mixed-order CAVs platoon. The velocity profiles are shown in Fig. 5, which indicates that velocity synchronization is realized within 0.5 *s* among all following vehicles with the leader. The acceleration curves are presented in Fig. 6, which demonstrates that the third-order following vehicles can quickly track the leader’s acceleration trajectory, while the second-order vehicles maintain consistent velocity without acceleration tracking requirements. This validates the effectiveness of the unified control framework for mixed-order dynamics. The spacing, velocity, and acceleration errors are shown in Figs. 7, 8 and 9, respectively. As we can see, the neighborhood synchronization errors $e_{p,i}$, $e_{v,i}$, and $e_{a,i}$ can converge to a bounded neighborhood of zero within 0.6 *s*, indicating that all following vehicles can track the leader’s velocity, while maintaining the desired inter-vehicle spacing. The small state errors confirm the satisfactory tracking performance of the proposed method under actuator faults and external disturbances. The estimated disturbance errors $\tilde{\varpi }_{i}$ are given in Fig. 10, where $\tilde{\varpi }_{i}$ converge to zero, which illustrates that the designed ADOB can effectively estimate $\varpi _i$.

To provide a deeper and more systematic analysis of how the critical design parameter *c* influences control system performance, Table 2 presents the root-mean-square errors (RMSEs) of the spacing errors $e_{p,i}$ and estimated disturbance errors $\tilde{\varpi }_i$ under different design parameters *c*. The RMSEs of $e_{p,i}$ and $\tilde{\varpi }_i$ are computed using the following formula

70 $$\begin{aligned} \textrm{RMSE}_{e_{p}}&\triangleq \frac{1}{5} \sum _{i=1}^{5} \sqrt{\frac{1}{N_\Bbbk } \sum _{\Bbbk =1}^{N_\Bbbk } \left( e_{p,i}(\Bbbk )\right) ^2} \nonumber \\ \textrm{RMSE}_{\tilde{\varpi }}&\triangleq \frac{1}{5} \sum _{i=1}^{5} \sqrt{\frac{1}{N_\Bbbk } \sum _{k=1}^{N_\Bbbk } \left( \tilde{\varpi }_i(\Bbbk )\right) ^2} \end{aligned}$$

where $\Bbbk$ is the time step, $N_\Bbbk$ represents the total number of time steps.

It can be observed from Table 2 that the design parameter *c* has opposite effects on the RMSEs of $e_{p,i}$ and $\tilde{\varpi }_i$, which requires us to select appropriate design parameter *c* according to the actual situation. The effectiveness and advantage of the proposed control method are verified.

**Table 2:** The RMSEs of $\tilde{\varpi }_i$ and $e_{p,i}$ under different design parameters *c*.

*c*	$\textrm{RMSE}_{e_{p}}$	$\textrm{RMSE}_{\tilde{\varpi }}$	*c*	$\textrm{RMSE}_{e_{p}}$	$\textrm{RMSE}_{\tilde{\varpi }}$
50	1.8739e-04	0.0399	500	1.8746e-04	0.0376
100	1.8740e-04	0.0313	550	1.8746e-04	0.0393
150	1.8741e-04	0.0290	600	1.8747e-04	0.0409
200	1.8742e-04	0.0290	650	1.8747e-04	0.0425
250	1.8743e-04	0.0300	700	1.8748e-04	0.0440
300	1.8743e-04	0.0312	750	1.8749e-04	0.0454
350	1.8744e-04	0.0330	800	1.8750e-04	0.0469
400	1.8745e-04	0.0343	850	1.8751e-04	0.0485
450	1.8745e-04	0.0363	900	1.8751e-04	0.0502

## Conclusion

This paper investigates the distributed adaptive fault-tolerant control problem for mixed-order CAVs platoon with actuator faults and saturation, external disturbances, and uncertainties. The studied mixed-order CAVs platoon consists of vehicles with mixed second- and third-order dynamics and all vehicles can have the different numbers and types of states. A novel ADOB is designed to approximate external disturbances as well as bias faults and improve the control performance of mixed-order CAVs platoon. The NNs-based adaptive mechanism is designed to approximate uncertainties and actuator efficiency factor, which can mitigate the adverse effects of uncertainties as well as actuator faults and enhance the system’s robustness. Based on the developed ADOB and NNs, a novel adaptive fault-tolerant control method for mixed-order (second- and third-order) CAVs platoon is proposed to ensure stability and achieve the control goals of the mixed-order CAVs platoon. The numerical example results can validate the effectiveness and advantage of the proposed control method. Building on this work, our future research will focus on developing reinforcement learning-based control strategies for mixed-order CAVs platoon to further enhance adaptability and decision-making capabilities under more complex and uncertain driving conditions (Fig. 8).

## Data Availability

All data are included in this published article.

## Code availability

The code is available from the corresponding author and can be given on request.
